# Optimized Electrochemical Sensor for Ochratoxin A Quantification in Coffee and Wheat Matrices

**DOI:** 10.1021/acsomega.5c03171

**Published:** 2025-06-26

**Authors:** Jane S. de Medeiros, Jairo P. Oliveira, Helder Knidel, Tárcila M. N. da Silva, José G. A. Rodrigues, Rafael Q. Ferreira

**Affiliations:** † Department of Chemistry/Center of Exact Sciences, 28126 Federal University of Espírito Santo, Fernando Ferrari Avenue, n° 514, Goiabeiras, Vitória, Espírito Santo 29075-910, Brazil; ‡ Department of Morphology/Health Sciences Center, Federal University of Espírito Santo, Marechal Campos Avenue, n° 1468, Maruípe, Vitória, Espírito Santo 29047-105, Brazil; § Kaffee Group, ES-146 Highway, km 6.1, Santa Maria, Marechal Floriano, Espírito Santo 29255-000, Brazil

## Abstract

Sensitive and selective ochratoxin A (OTA) detection in challenging food matrices (wheat, coffee) is achieved using a cost-effective electrochemical method. The approach utilizes commercial carbon black-graphite screen-printed electrodes (SPE CB-G) enhanced via *in situ* modification with cetyltrimethylammonium bromide (CTAB). Adsorptive stripping differential pulse voltammetry (AdSDPV) parameters are rigorously optimized using central composite design (CCD), resulting in an optimal deposition time of 205 s and a CTAB concentration of 80 μmol L^–1^. The method achieves a limit of detection (LOD) of 1.39 ng mL^–1^ and a limit of quantification (LOQ) of 4.20 ng mL^–1^ in 0.2 mol L^–1^ phosphate buffer (pH 7.0) solution. In roasted and ground Arabica coffee (*Coffea arabica*), the method demonstrates a LOD of 2.70 ng mL^–1^ and a LOQ of 8.20 ng mL^–1^, with recovery rates ranging from 94.64 to 109.86%, compliant with key regulatory standards. The presence of CTAB minimizes the matrix effects and ensures reliable and reproducible results. This optimized SPE-based sensor offers a practical and efficient alternative to conventional methods for routine food safety monitoring.

## Graphical abstract

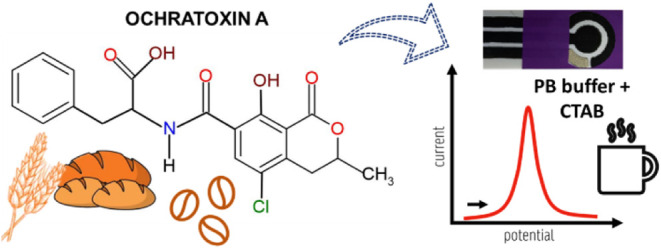

## 1 Introduction

Ochratoxin A (OTA), a mycotoxin of global significance, is predominantly synthesized by fungi belonging to the genera *Aspergillus* and *Penicillium*. This toxin is commonly found in various food items, such as grains and grain-based products, dehydrated fruits, herbs and spices, ground and whole bean coffee, chocolate-derived goods, and other foodstuffs.^1–3^ Long-term exposure to OTA has been associated with serious adverse health outcomes, including kidney and liver damage, birth defects, and possible cancer-causing effects.^1,4^ Furthermore, research indicates a potential correlation between OTA and Balkan endemic nephropathy (BEN) and the occurrence of neoplasms in the urinary tract.^4^ In 1993, the International Agency for Research on Cancer (IARC) categorized OTA as a Group 2B substance, classifying it as a possible carcinogen for humans.^5^

Given the health risks associated with OTA, regulatory authorities worldwide have established maximum allowable levels of this mycotoxin in food. These regulations aim to mitigate human and animal exposure, primarily through the ingestion of contaminated products, which poses significant public health challenges.^2,6,7^

In Brazil, the National Health Surveillance Agency (Anvisa) has set specific OTA limits for various food categories, such as 10 μg kg^–1^ for processed cereals and cereal-based products, 20 μg kg^–1^ for fresh cereals, 10 μg kg^–1^ for roasted and instant coffee, 30 μg kg^–1^ for spices, and 10 μg kg^–1^ for dried and dehydrated fruits.^8^ The European Union has implemented stricter regulations, with limits ranging from 5.0 μg kg^–1^ for unprocessed cereal grains and soluble coffee, 3.0 μg kg^–1^ for roasted coffee, 15–20 μg kg^–1^ for dried peppers and spices, and from 2.0 to 8.0 μg kg^–1^ for dried and dehydrated fruits.^9^

To address the challenges of OTA detection and control, understanding its physicochemical properties is essential, as they influence its behavior in biological and analytical systems.^4,6,10,11^ Structurally, OTA is a weak organic acid comprising a dihydroisocoumarin group that is covalently bonded to a l-β-phenylalanine molecule via an amide bond.^7,10,12,13^ OTA, with a molecular formula of C_20_H_18_ClNO_6_ and a molar mass of 403.8 g mol^–1^, appears as a white, odorless crystalline solid with a melting point between 169 and 173 °C. It is poorly soluble in water, but readily dissolves in polar organic solvents and alkaline solutions.^2,6,14,15^

In an aqueous solution, OTA exhibits a complex acid–base behavior, characterized by two dissociation constants: p*K*_a1_ ≈4.2–4.4 (carboxylic group of phenylalanine) and p*K*_a2_ ≈7.0–7.3 (phenolic hydroxyl group).^4,10,16^ Under alkaline conditions (pH > 10), OTA undergoes lactone ring opening and hydrolysis, although these processes make p*K*_a_ determination challenging.^10,16^ These physicochemical properties are critical for understanding the stability and behavior of OTA in various food matrices and detection systems because they influence its interactions with matrix components and analytical responses.^10,12,13,17–19^

The detection and quantification of OTA in food present a significant analytical challenge owing to its low concentration and the complexity of food matrices. Traditionally, chromatographic techniques such as high-performance liquid chromatography (HPLC) with fluorescence detectors (FLD) or tandem mass spectrometry (MS/MS) have been extensively utilized owing to their high sensitivity and selectivity.^2,14,20,21^ However, these methods have limitations, including complex sample preparation, use of toxic organic solvents, high equipment costs, lengthy analysis times, and considerable environmental impact.^20–22^

Immunological methods, such as enzyme-linked immunosorbent assay (ELISA) and lateral flow immunochromatographic assay (LFIA), provide rapid and simplified alternatives. Nevertheless, these techniques may be susceptible to interference and require meticulous handling of reagents and antibodies.^21,23–25^

Electroanalytical methods, particularly voltammetry, have emerged as promising and cost-effective alternatives for OTA detection. These methods offer advantages such as lower acquisition and maintenance costs, ease of operation, rapid response times, and high specificity and sensitivity, with limits of detection (LOD) reaching the ng mL^–1^ level, and in some cases the pg mL^–1^ level.^12,20,26–30^ The miniaturization of electroanalytical systems further enhances their practicality compared to chromatographic and immunoassay-based approaches, especially when combined with portable potentiostats and unmodified screen-printed electrodes (SPEs).^12,20,26–29,31^ The field continuously explores novel materials, including advanced macrocyclic catalysts like phthalocyanines^32^ and nanomaterial-based macromolecule-nanoparticle hybrids,^33^ which have significantly expanded the sensitivity and versatility of electrochemical sensors for diverse analytical applications, as highlighted in recent reviews.^32,33^

Among voltammetric techniques, differential pulse voltammetry (DPV) is notable for its high resolution and sensitivity for detecting irreversible redox processes. Adsorptive stripping differential pulse voltammetry (AdSDPV) is effective for OTA detection when combined with cationic surfactants such as cetyltrimethylammonium bromide (CTAB). CTAB enhanced the electrochemical response by promoting OTA adsorption onto the electrode surface through hydrophobic and electrostatic interactions. This adsorption increased the peak current intensity, thereby improving the analyte detectability.^19,30,34–37^

Despite the growing interest in electroanalytical detection of OTA, few studies have comprehensively addressed the combined effects of electrode surface composition and surfactant-mediated adsorption on the voltammetric performance in real food matrices. Most existing works are limited to laboratory conditions or involve complex electrode modifications that hinder scalability. This study addresses this critical gap by developing and optimizing a simple, cost-effective, and highly sensitive electrochemical method for OTA detection in complex matrices such as roasted coffee and wheat. The method leverages the synergistic properties of carbon black-graphite screen-printed electrodes (SPE CB-G) and *in situ* modification with CTAB, enhanced through a chemometric optimization via central composite design (CCD).

## 2 Experimental Section

### 2.1 Reagents and Solutions

All solutions were prepared using analytical-grade reagents and ultrapure water obtained from a purification system (H2O MA-UV-T, Sartorius Arium Mini, Germany) with a resistivity of 18 MΩ cm at 25 °C. A phosphate buffer solution (PB) at 0.2 mol L^–1^ (pH 6.0–8.0) was prepared using anhydrous monobasic potassium phosphate (99%, Neon, Brazil) and anhydrous dibasic potassium phosphate (99%, J.T. Baker, Mexico). To prepare the acetate buffer (ACE) at 0.25 mol L^–1^ (pH 3.0–5.0), acetic acid (99.7%, Vetec, Brazil) and sodium acetate trihydrate (99.5%, Riedel-de Haën, Germany) were used. The pH was adjusted using hydrochloric acid (37%, Neon, Brazil) or sodium hydroxide (97%, Proquímios, Brazil) at a concentration of 1.0 mol L^–1^.

CTAB (98%, Sigma-Aldrich, Germany) was prepared at a concentration of 0.154 g in 10.0 mL ultrapure water. A stock solution of OTA was prepared from a standard derived from *Petromyces albertensis* [HPLC grade] (98%, Sigma-Aldrich, USA) at a concentration of 1.0 mg mL^–1^ (2.48 mmol L^–1^) in anhydrous ethanol (99.8%, Dinâmica, Brazil). This solution was aliquoted into 10 portions of 100 μL and stored in 200 μL microtubes at −4 °C in the freezer compartment, protected from light to prevent its degradation.

For the interference studies, solutions of caffeine (1.95 mg mL^–1^), chlorogenic acid (0.5 mg mL^–1^), furfural (1.0 mg mL^–1^), trigonelline (0.5 mg mL^–1^), caffeic acid (10 mg mL^–1^), and acrylamide (0.01 mg mL^–1^) were prepared in methanol.

### 2.2 Equipment and Apparatus

All reagents and samples were weighed using an analytical balance with a capacity of 220 g (NS A017153; Edutec, Brazil). Electroanalytical measurements were performed using a portable μStat 400 potentiostat/galvanostat (Metrohm DropSens, Spain) operated with an updated version of the proprietary software DropView 8400 M (version 1.28, Metrohm DropSens, Spain) on a computer running the Windows 11 operating system.

Electrochemical analysis of the OTA standard solutions was initially performed, followed by the analysis of wheat and Arabica coffee samples using a commercial SPE system. The SPEs employed were of type C2 (ADB Sensores, Brazil), composed of a working electrode with a diameter of 4 mm, carbon black (CB), and graphite (G) particles finely dispersed in varnish and solvents (SPE CB-G). The SPE also included a counter electrode made of the same material and an Ag/AgCl pseudoreference electrode, all of which were deposited on an acetate substrate. A compatible connector cable from ADB Sensors was used in conjunction with the Metrohm DropSens system to connect the SPE to the potentiostat.

### 2.3 Surface Characterization

Raman spectra and atomic force microscopy (AFM) surface analyses were conducted to characterize the SPE CB-G surface before and after CTAB modification. Raman spectra were recorded using a confocal Raman-AFM microscope (WITec alpha300 RA, Germany) equipped with a Zeiss EC Epiplan lens and a 532 nm excitation laser. Acquisition parameters included a 600 lines/mm grating (BLZ = 500 nm), 200 μm confocal aperture, spectral range from 150 to 3750 cm^–1^, and 30 accumulations with 0.5 s each. Optical micrographs (60 μm window) were also obtained using the same system.

For AFM analysis, topographical 3D surface images were obtained simultaneously using the same WITec instrument, operating in noncontact mode, over representative 20.0 μm × 20.0 μm areas of the electrode surface. Roughness parameters, such as average roughness (*S*_a_) and root-mean-square roughness (*S*_q_), maximum peak-to-valley height (Sz), and developed surface area, were determined from the AFM topography (height) data using the Gwyddion open-source software.

### 2.4 Electrochemical Measurements

#### 2.4.1 Electrode Preparation

Before the analysis of the voltammetric profile of OTA by cyclic voltammetry (CV), electrochemical stabilization of the SPE was performed using CV. This stabilization was conducted in the potential range of +0.05 to +1.15 V, with a scan rate (ν) of 50 mV s^–1^, step potential of 2 mV, and five successive scans. During this process, 80 μL aliquots of 0.25 mol L^–1^ ACE buffer (pH 3.0–5.0) and 0.2 mol L^–1^ PB buffer (pH 6.0–8.0) were applied to the SPE area corresponding to the electrochemical cell.

The SPE electrochemical parameters were adjusted for studies involving the CV scan rate, DPV, and AdSDPV. In these cases, the potential range was narrowed to +0.1 to +0.9 V, maintaining ν of 50 mV s^–1^, a step potential of 2 mV, and five successive scans. The stabilization of the SPE was performed by cyclic voltammetry using a 0.2 mol L^–1^ PB buffer (pH 7.0) for the CV scan rate and DPV studies. For the CV scan rate with CTAB and AdSDPV studies and subsequent assays, electrochemical treatment with surface modification was performed using a 0.2 mol L^–1^ PB buffer (pH 7.0) with CTAB to enhance the electrode response. The experiments were conducted in an electrochemical cell adapted to hold 5 mL of the solution, with the SPE immersed until the entire active area of the electrode was in contact with the solution. At each step, the solution was magnetically stirred for 30 s at 500 rpm (HJ-4; Even Brazil). All electroanalytical measurements were performed in the presence of dissolved oxygen.

The anodic peak current (*I*_p_) corresponding to OTA oxidation obtained by CV was measured directly using a potentiostat with proprietary software. Manual measurements were performed according to the manufacturer’s guidelines if the software could not automatically measure *I*_p_. For pulse voltammetry techniques, the anodic *I*_p_ height was determined after baseline correction using the potentiostat’s proprietary software.

#### 2.4.2 Electrochemical Impedance Spectroscopy (EIS)

EIS measurements were performed using an Autolab PGSTAT128N potentiostat/galvanostat (Metrohm, Switzerland), with data processing conducted via Nova 2.1.8 software (Metrohm). Measurements were carried out at a DC potential of +0.70 V (*vs pseudo* Ag/AgCl), corresponding to the approximate oxidation potential of OTA under these experimental conditions, with an AC sinusoidal perturbation amplitude of 0.01 V_RMS_ across a frequency range of 100 kHz to 0.01 Hz (5 points per decade). All experiments were conducted in an electrochemical cell containing 0.2 mol L^–1^ PB buffer (pH 7.0) spiked with 25 μmol L^–1^ OTA. Two experimental conditions were evaluated: (i) without CTAB and (ii) with 80 μmol L^–1^ CTAB. For each condition, a new SPE was used. Before EIS analysis, the SPEs were electrochemically stabilized by CV (as detailed in Section 2.4.1) in either PB (pH 7.0) for condition (i) or PB (pH 7.0) containing 80 μmol L^–1^ CTAB for condition (ii). Impedance data were fitted to an equivalent electrical circuit using Nova 2.1.8 software to determine the charge transfer resistance (*R*_*ct*_).

#### 2.4.3 Voltammetric Profile Study

The electrochemical behavior of OTA was investigated using CV under various pH values. Aliquots of 80 μL of OTA solution, prepared at a concentration of 10.0 μmol L^–1^, were analyzed in 0.25 mol L^–1^ ACE buffer at pH 3.0–5.0 and 0.2 mol L^–1^ PB buffer at pH 6.0–8.0. A new SPE was employed under each pH condition to ensure reproducibility. The CV parameters included a potential range of +0.05 to +1.15 V, a ν of 50 mV s^–1^, a step potential of 2 mV, and a single scan per measurement. Between each scan, a droplet of 80 μL was replaced with a fresh aliquot of OTA-containing solution.

To evaluate the effect of the scan rate on the voltammetric response of OTA, additional experiments were performed in 0.2 mol L^–1^ PB buffer (pH 7.0), with scan rates ranging from 25 to 350 mV s^–1^. A complementary study was conducted to assess the influence of CTAB on OTA oxidation. CTAB was added to a 0.2 mol L^–1^ PB buffer at a final concentration of 80 μmol L^–1^. A deposition potential (*E*_dep_) of +0.55 V was applied to the SPE for 205 s with continuous stirring to enhance OTA adsorption onto the electrode surface. After a 10 s resting period, voltammetric measurements were performed.

In both studies, CV measurements were performed in the potential range of +0.10 to +0.90 V, with a step potential of 2 mV and a single scan per measurement. The solution was stirred for 30 s between scans to promote homogeneity. These experiments provided insights into the pH-dependent electrochemical behavior of OTA, the effect of the scan rate on its response, and the role of CTAB in enhancing oxidation signals.

#### 2.4.4 Pulse Voltammetry and Optimization

To optimize the parameters for DPV and AdSDPV, a CCD was employed, considering two variables (*k* = 2).^28,38–41^ For DPV, the optimization focused on the pulse potential (*E*_pulse_, mV) and pulse time (*t*_pulse_, ms). The levels tested for each variable are listed in Table S1. Experimental parameters, such as a ν of 10 mV s^–1^ and a step potential of 5 mV, were established based on previous studies.^12,19,27,28,42^ The applied potential range was from +0.40 to +0.90 V, and the OTA concentration used in these experiments was 5.0 μmol L^–1^, prepared in 0.2 mol L^–1^ PB buffer at pH 7.0 in an adapted 5 mL electrochemical cell.

For AdSDPV, the optimized parameters included the deposition time (*t*_dep_, s) and CTAB concentration (μmol L^–1^). The levels and designs of the variables are presented in Table S2. A fixed *E*_dep_ of +0.55 V was selected based on previous studies.^19,42^ The OTA concentration used in this set of experiments was 201.91 ng mL^–1^ (0.5 μmol L^–1^).

The CCD model consisted of 13 experiments, including five replicates at the central point of the model. This experimental design facilitated the evaluation of the influence of *E*_pulse_, *t*_pulse_, *t*_dep_, and CTAB concentration on the anodic *I*_p_ response for OTA oxidation. Statistical analysis of the experimental results, as well as the generation of response surface plots and graphical representations of *I*_p_, were performed using the design of experiments (DoE) application integrated into Origin 2024 Professional software (version 2024b). A 95% confidence interval was applied to all analyses to ensure robust and reliable conclusions.

### 2.5 Sample Preparation

Ground wheat samples were obtained from a local commercial supplier. Sample preparation for AdSDPV analysis was performed as follows: 2.5 g of wheat sample was weighed and mixed with 12.5 mL of 0.2 mol L^–1^ PB buffer pH 7.0, followed by manual agitation for 10 min. The mixture was centrifuged at 2000 rpm for 5 min (80–2 B; EDUTEC, Brazil). The supernatant was passed through a syringe filter (Millex PVDF, Germany) with a pore size of 0.45 μm and a diameter of 33 mm to remove fine suspended solids. The filtered wheat extract was stored in a Falcon tube at approximately 5 °C and analyzed within 1 week of preparation. For voltammetric analysis, 0.5 mL (500 μL) of the filtered wheat extract was added to 5 mL of 0.2 mol L^–1^ PB buffer pH 7.0 containing CTAB.

A sample of roasted and ground Arabica coffee beans was prepared using clean, dried beans free of impurities, such as husks, leaves, wood fragments, broken beans, moldy beans, or pest-damaged beans. The beans were roasted at 180 °C (medium roast) for 9 min and ground to a particle size suitable for filtered coffee (particle size no. 49). The test solution (2.5 g) was dissolved in 25 mL of 0.2 mol L^–1^ PB buffer (pH 7.0). The mixture was stirred using a vortex mixer (QL-901, Edulab, Brazil) for 5 min, followed by filtration through a commercial coffee filter (No. 102, Três Corações, Brazil) to remove larger particulates. The resulting filtrate was passed through a syringe filter (Millex PVDF, Germany) with a pore size of 0.45 μm and a diameter of 33 mm to eliminate the fine suspended solids. The final solution was stored in a 50 mL Falcon tube at approximately 5 °C and used for subsequent analyses within 1 week. For voltammetric analysis, 0.1 mL (100 μL) of the filtered coffee extract was mixed with 5 mL of 0.2 mol L^–1^ PB buffer (pH 7.0) containing CTAB.

### 2.6 Calibration Curve, Interferences, and Matrix Effect

A calibration curve for OTA was constructed over a concentration range of 10.09 to 403.81 ng mL^–1^ (25 nmol L^–1^ to 1 μmol L^–1^) in 0.2 mol L^–1^ PB buffer at pH 7.0, supplemented with 80 μmol L^–1^ CTAB. The voltammetric technique employed was AdSDPV, with optimized parameters obtained through CCD, as summarized in Table 1, which presents the optimized parameters for DPV and AdSDPV obtained using CCD: a potential window of +0.55 to +0.90 V, a scan rate of 10 mV s^–1^, a step potential of 5 mV, a deposition time of 205 s, and a stirring rate of 500 rpm.

**Table 1:** Parameters Optimized for the DPV and AdSPDV Techniques Were Obtained Using CCD

Parameter^a^	**DPV**	**AdSDPV**
Potential range	+0.40 to +0.90 V	+0.55 to +0.90 V
Scan rate (ν)	10 mV s^–1^	10 mV s^–1^
Step potential	5 mV	5 mV
Pulse potential (*E*_pulse_)	100 mV	100 mV
Pulse time (*t*_pulse_)	25 ms	25 ms
Deposition time (*t*_dep_)	N/A	205 s
Stirring rate	N/A	500 rpm
Deposition potential (*E*_dep_)	N/A	+0.55 V
CTAB (μmol L^–1^)	N/A	80
Equilibration time	10 s	10 s

a This table summarizes the optimized electrochemical parameters obtained through CCD for OTA determination using the DPV and AdSDPV techniques.

All analytical measurements (calibration curves, recovery tests) were performed in triplicate (*n* = 3) to ensure robustness. However, the measurement protocol differed between standard solutions and matrix extracts. For measurements in buffer (e.g., calibration curve, recovery in buffer), three consecutive scans were recorded using the same SPE for each concentration point or replicate, and the average *I*_p_ was used. This approach was validated by repeatability tests (Section 3.6 and Figure S11). Conversely, for analyses involving wheat and coffee extracts, significant signal attenuation was observed after the first voltammetric scan, likely due to strong matrix adsorption or electrode fouling. Therefore, to ensure data reliability in these complex matrices, a new SPE was used for each replicate measurement, and only the *I*_p_ from the first scan was considered for calculations.

An interference study was conducted exclusively on the coffee matrix to evaluate the impact of common coffee components, such as caffeine, chlorogenic acid, furfural, trigonelline, caffeic acid, and acrylamide. Each interferent was added to an electrochemical cell containing 5 mL of OTA solution at a fixed concentration of 201.91 ng mL^–1^(0.5 μmol L^–1^). The final concentrations of the interferents (Table S3) ranged from 100 ng mL^–1^ (acrylamide) to 19,500 ng mL^–1^ (caffeine). These levels were estimated to represent realistic concentrations after considering typical values reported in the literature for these compounds in roasted coffee and accounting for the 1:50 dilution of the initial coffee extract in the electrochemical cell (see Section 2.4).^43–45^ The resulting proportions relative to the fixed OTA concentration (201.91 ng mL^–1^) are listed in Table S3.

Additionally, the effect of the coffee matrix was evaluated by introducing a 100 μL aliquot of the prepared coffee solution into an electrochemical cell containing 5 mL of 0.2 mol L^–1^ PB buffer (pH 7.0) supplemented with 80 μmol L^–1^ CTAB with OTA at a concentration of 201.91 ng mL^–1^. A calibration curve was constructed in the presence of a coffee matrix to evaluate the feasibility of the developed method under conditions that approximate real-world analytical scenarios.

## 3 Results and Discussion

### 3.1 Surface Characterization of Bare and CTAB-Modified SPE CB-G

To investigate the surface characteristics of the SPEs before and after *in situ* modification with CTAB, Raman spectroscopy and AFM were employed.

#### 3.1.1 Raman Spectroscopy

Optical micrographs obtained via the Raman microscope showed a typical heterogeneous surface characteristic of carbon-based composite materials for both bare and CTAB-modified SPEs, with no visually distinct large-scale changes upon CTAB modification at this magnification (Figure S1).

Raman spectra (Figure S2) exhibited prominent D (∼1345 cm^–1^), G (∼1580 cm^–1^), and 2D (∼2700 cm^–1^) bands in both modified and unmodified electrodes, typical of carbonaceous materials. The intensity ratio of the D band to the G band (*I*_D_ */I*_G_) is often used to characterize the degree of disorder or defect density in carbon materials.^27,46,47^ For the bare SPE CB-G, the *I*_D_ */I*_G_ ratio was calculated to be 0.27. After modification with CTAB, this ratio was found to be 0.27. The relative intensities of the D and G bands (*I*_D_/*I*_G_ ratio) remained comparable, indicating that the structural integrity of the carbonaceous material was preserved upon CTAB adsorption. However, a slight broadening and intensity reduction in the CTAB-modified sample suggest an increase in surface coverage and changes in surface energy due to surfactant adsorption.

#### 3.1.2 Atomic Force Microscopy (AFM)

The surface morphology of bare and CTAB-modified SPE CB-G electrodes was analyzed by AFM in topographical mode over representative 20.0 μm × 20.0 μm areas. The bare SPE CB-G surface (Figure S3a) exhibits a heterogeneous, rough surface, typical of aggregated carbon particles and binder components. After *in situ* modification with CTAB (Figure S3b, the surface topography appears notably smoother and more uniform, suggesting the formation of a CTAB layer that covers the underlying electrode structure and potentially fills in some of the crevices or pores.

Quantitative statistical analysis of AFM topography confirmed these morphological changes. The *S*_a_ decreased markedly from 1.53 μm (unmodified SPE) to 0.97 μm (CTAB-modified SPE), and *S*_q_ similarly decreased from 1.96 to 1.33 μm. Concurrently, the maximum peak-to-valley height (*S*_*z*_) was reduced from 10.22 (for the bare electrode) to 7.22 μm (for the CTAB-modified surface). Furthermore, the developed surface area (for the 400 μm^2^ projected area) decreased from 1350.45 (for the bare SPE) to 1167.89 μm^2^ (after CTAB modification). These changes reflect substantial smoothing and homogenization of the electrode surface upon CTAB adsorption.

The AFM results quantitatively demonstrate that CTAB modification homogenizes the SPE CB-G surface, forming a smoother and more uniform film. This morphological change creates a well-defined, stable electrochemical interface, which enhances reproducibility, reduces capacitive background current, and optimizes the conditions for OTA interaction and detection.

### 3.2 Electrochemical Behavior of OTA

The electrochemical oxidation of OTA on the SPE was investigated by CV, evaluating the anodic peak potential (*E*_p_) and anodic peak current (*I*_p_) under different pH conditions using 0.25 mol L^–1^ ACE buffer (pH 3.0–5.0) and 0.2 mol L^–1^ PB buffer (pH 6.0–8.0). The cyclic voltammograms presented in Figure 1 demonstrate that the electrochemical profile of OTA was highly dependent on the pH of the supporting electrolyte.

**Figure 1:**
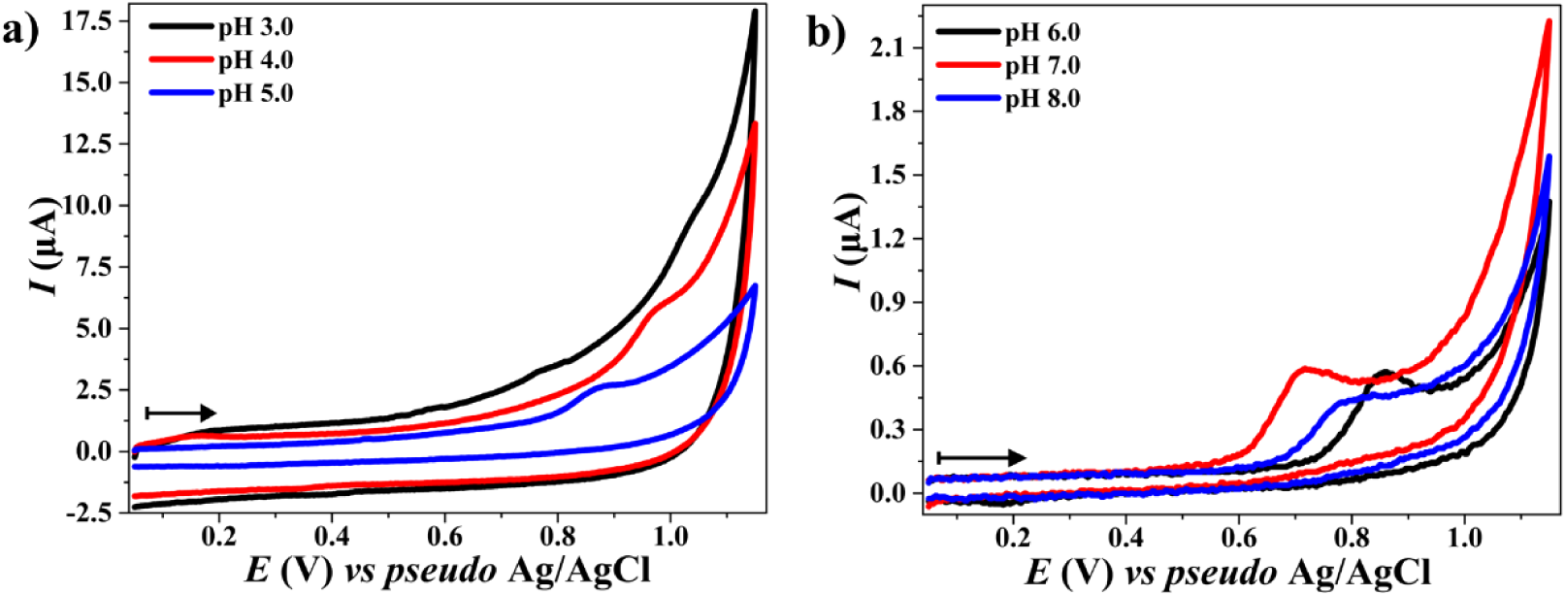
Cyclic voltammograms of OTA oxidation at different pH values. (a) 0.25 mol L^–1^ ACE buffer (pH 3.0–5.0): black, pH 3.0; red, pH 4.0; and blue, pH 5.0. (b) 0.2 mol L^–1^ PB buffer (pH 6.0–8.0): black, pH 6.0; red, pH 7.0; and blue, pH 8.0. The OTA concentration was 10 μmol L^–1^. The arrows indicate the scanning direction. The scan rate was set to 50 mV s^–1^.

In the analysis performed using 0.25 mol L^–1^ ACE buffer (pH 4.0) (Figure 1a), the anodic *E*_p_ was observed at +0.97 V (*vs pseudo* Ag/AgCl) during the first scan, a significantly higher value compared to that obtained in a neutral to slightly alkaline medium, such as 0.2 mol L^–1^ PB buffer at pH 7.0 (Figure 1b), whereas anodic *E*_p_ was recorded at +0.72 V (*vs pseudo* Ag/AgCl). The lower background current for OTA oxidation in PB buffer (0.2 mol L^–1^, pH 7.0) compared to ACE (0.25 mol L^–1^, pH 4.0) stems from PB’s higher ionic strength, which reduces double-layer capacitance. Additionally, PB’s neutral pH minimizes non-Faradaic currents, allowing for clearer OTA oxidation signals. These properties, combined with the preferential deprotonation of OTA at pH 7.0, result in more intense and better-defined oxidation signals, as demonstrated in the voltammograms of Figure 1b. These results indicate that OTA oxidation occurs at less positive potentials at slightly alkaline pH, which aligns with the expected variations owing to changes in the protonation/deprotonation state of OTA at different pH values (Figure S4).^10,12,13,17–19,48^ The literature reports that the anodic *E*_p_ for OTA varies between +0.80 and +1.20 V, depending on the analyte concentration, the pH of the electrolyte, and the type of working and reference electrode used.^12,13,49^ The values obtained for SPE were consistent with those reported in the literature, reinforcing its effectiveness as a detection system for OTA.^12,13,49^

### 3.3 Irreversibility and Adsorption Effect

The behavior of OTA during electrochemical processes reveals its oxidative irreversibility and adsorptive effects. Under acidic conditions (Figure 2a), a distinct cathodic *E*_p_ value suggests the reduction of oxidized OTA to its ochratoxin quinone (OTQ).^11–13,49^ However, at neutral to alkaline pH (Figure 2b), the absence of such peaks indicates increased irreversibility in the oxidation process. The oxidative process also resulted in a gradual decline in the anodic *I*_p_ during successive scans, which was attributed to the formation of an adsorptive film from OTA oxidation products. This film partially inhibits the active electrode surface, thereby hindering electron transfer. The redox couple OTQ/OTHQ (OTHQ, ochratoxin hydroquinone form) contributed to the adsorptive behavior, whereas the irreversible oxidation of OTHQ resulted in OTCT (ochratoxin catechol form) compounds, which caused the current decline. This dual interaction between irreversible oxidation and surface adsorption underscores the complexity of the electrochemical behavior of OTA, which could have implications for sensor design and optimization in analytical applications.^11–13,49^

**Figure 2:**
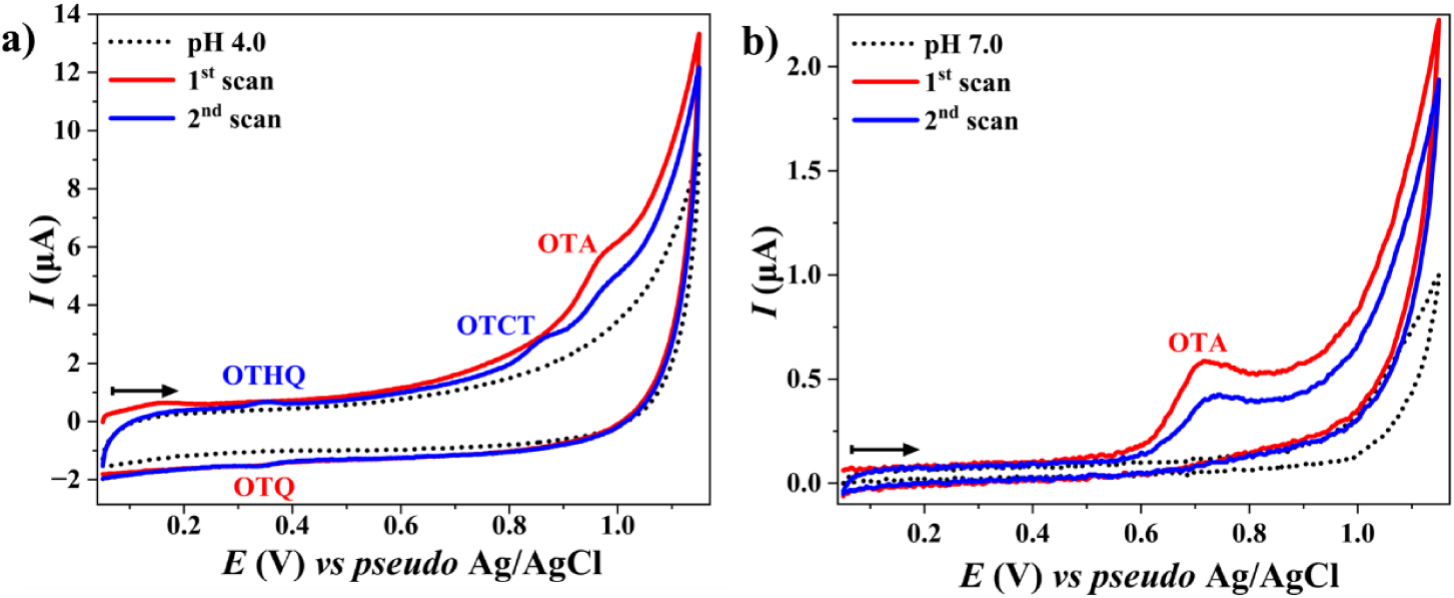
Cyclic voltammograms of OTA oxidation in (a) 0.25 mol L^–1^ ACE buffer at pH 4.0 and (b) 0.2 mol L^–1^ PB buffer at pH 7.0. OTA concentration: 10 μmol L^–1^. The black dotted lines represent voltammograms without OTA, and the red and blue lines correspond to the first and second scans, respectively, in the presence of OTA. The arrows indicate the scanning direction. The scan rate was set to 50 mV s^–1^.

Furthermore, the irreversibility of the redox process was confirmed, as evidenced by the difference in potential (Δ*E*_p_), which exceeded 59 mV for all pH values. For instance, in 0.2 mol L^–1^ PB buffer at pH 7.0, Δ*E*_p_ was +0.72 V, clearly indicating an irreversible redox process owing to slow electron transfer kinetics between the analyte and the electrode surface, as defined by the electrochemical irreversibility criteria.^30,50^

### 3.4 pH Influence on Anodic *E*_p_ and *I*_p_

The relationship between anodic *E*_p_ and pH is shown in Figure 3. The anodic *E*_p_ shifted linearly toward less positive values as the pH increased, following the relationship *E*_p_ (V) = −0.058 (pH) + 1.194 (*r* = −0.932; *R* ^2^ = 0.869). The slope at 58 mV pH^–1^ closely aligns with the theoretical Nernstian value of 59 mV pH^–1^, suggesting that OTA oxidation involves the transfer of one proton and one electron.^12,27,28^

**Figure 3:**
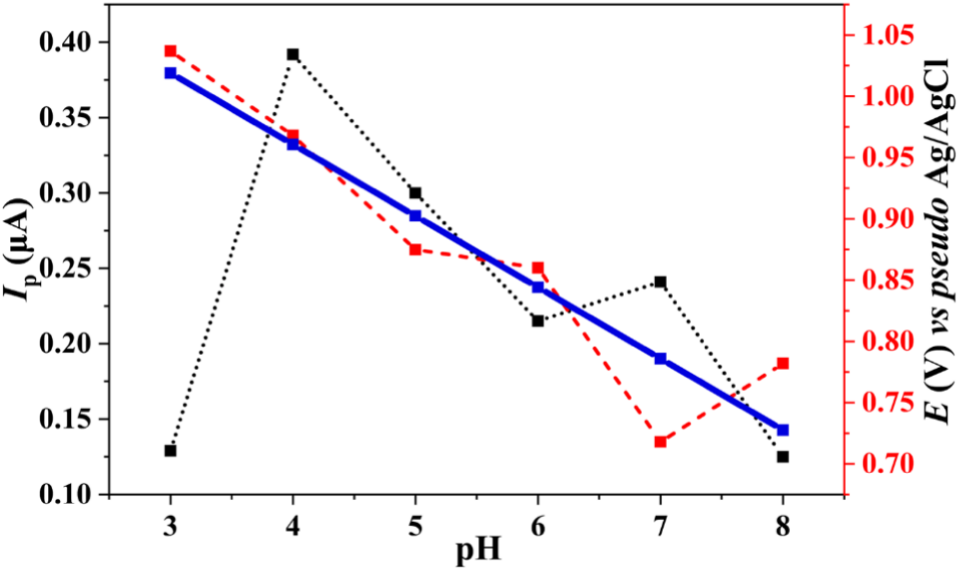
Influence of pH on anodic *I*_p_ and anodic *E*_p_ for OTA oxidation in 0.25 mol L^–1^ ACE buffer (pH 3.0–5.0) and 0.2 mol L^–1^ PB buffer (pH 6.0–8.0). OTA concentration: 10 μmol L^–1^. The black points and red dashed lines represent the variations in anodic *I*_p_ and *E*_p_, respectively, as a function of the pH. The blue lines indicate the linear regression of anodic *E*_p_ as a function of pH, described by the equation *E*_p_ (V) = – 0.058 (pH) + 1.194 (*R* ^2^ = 0.869).

The anodic *I*_p_ was also influenced by the pH of the medium (Figure 3). Generally, an increase in the anodic *I*_p_ was observed with increasing pH up to an optimum value, after which the current decreased. This behavior can be attributed to changes in the solubility and ionic form of OTA at different pH levels. For instance, in 0.25 mol L^–1^ ACE buffer at pH 4.0, the anodic *I*_p_ was 0.39 μA, whereas in 0.2 mol L^–1^ PB buffer at pH 7.0, the *I*_p_ was 0.24 μA, showing a slight increase after the decline observed at pH 5.0 and 6.0.

These results emphasize the significant influence of pH on the electrochemical oxidation of OTA. The observed anodic potential shift toward lower values with increasing pH is consistent with the deprotonation of OTA functional groups, in agreement with its known p*K*_a_ values (Figure S1).^10,13,17,19,48^ Studies suggest that OTA oxidation involves two protons and one electron (2H^+^/1e^–^) at pH below 7.0 and one proton and one electron (1H^+^/1e^–^) at pH 7.0. Under alkaline conditions, the process is independent of the pH and involves the transfer of a single electron (1e^–^).^12,13,49^ The final product of OTA oxidation is quinone, and its formation involves nucleophilic water addition followed by chloride ion release.^12,13,49^ The proposed mechanism of OTA oxidation is illustrated in Figure 4.^11,19,27^

**Figure 4:**
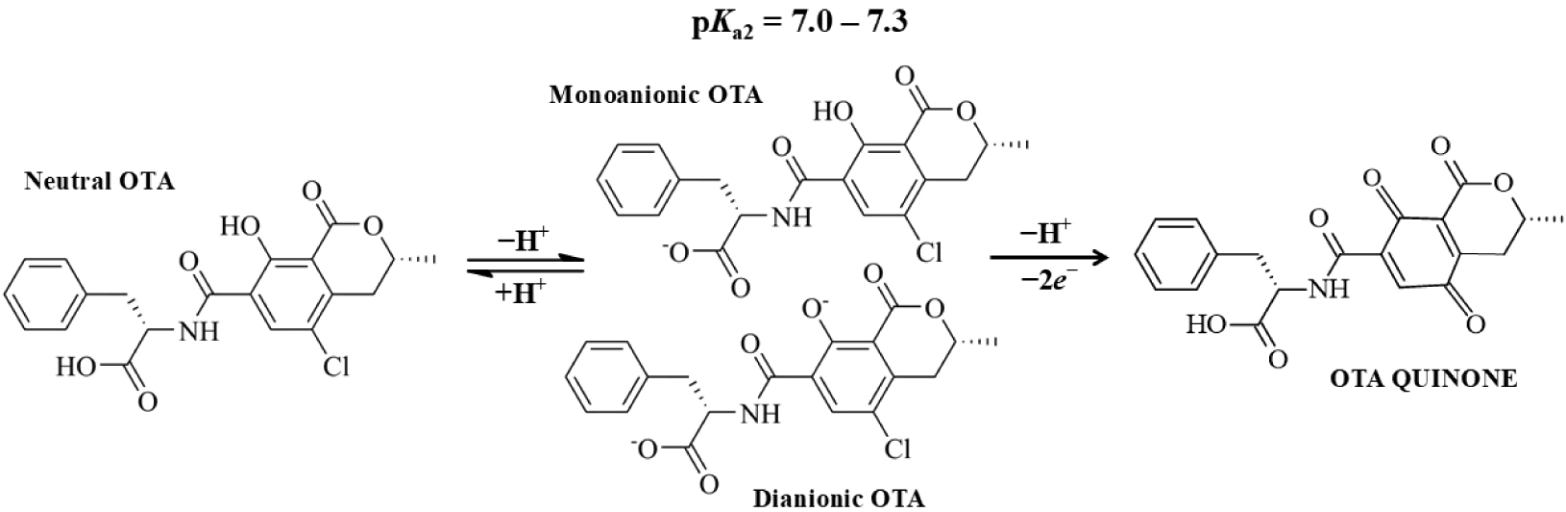
Proposed mechanism of OTA electrooxidation. At pH 7.0, the monoanionic and dianionic forms of OTA prevail, and oxidation involves the loss of one proton and two electrons, resulting in the formation of quinone with the participation of a water molecule and the release of chloride ions.

These findings highlight the critical role of pH in the electrochemical behavior of OTA and reinforce the relevance of the PB buffer at pH 7.0 for subsequent analysis. This electrolyte provides a lower oxidation potential and greater stability of voltammetric signals on the SPE, making it an optimal choice for the development of electroanalytical methods for OTA detection with minimal electrolyte decomposition in the applied potential range.

### 3.5 Influence of Scan Rate on OTA Oxidation

The influence of scan rate on OTA oxidation was investigated by CV under two different conditions: without CTAB (in 0.2 mol L^–1^ PB buffer pH 7.0) and with CTAB (in 0.2 mol L^–1^ PB buffer pH 7.0).

#### 3.5.1 OTA Oxidation in PB pH 7.0 (without CTAB)

Cyclic voltammograms (Figure S5a) recorded at scan rates ranging from 25 to 350 mV s^–1^ in 0.2 mol L^–1^ PB buffer (pH 7.0) revealed a progressive increase in anodic *I*_p_ and a slight anodic shift in *E*_p_ from +0.642 to +0.661 V. This behavior is typical of kinetically slow, irreversible oxidation processes, where higher scan rates accentuate the electron transfer limitation.^12,30^

At first glance, the plot of *I*_p_ *versus* the square root of the scan rate (ν^1/2^) exhibited a strong linear correlation (*R* ^2^ = 0.947), as shown in Figure S5b. This is characteristic of a diffusion-controlled^12,13,28^ electrochemical process and aligns with the Randles–Ševčík relationship for irreversible systems: *I*_p_ = (2.99 × 10 ^5^ *n* (*αn’*)^1/2^ *A C D*_ο_^1/2^ ν^1/2^, where *I*_p_ is the peak current (A), *n* is the total number of electrons involved in the reaction, α is the charge transfer coefficient, *n’* is the number of electrons transferred in the rate-determining step, *A* is the geometric area of the electrode (cm^2^), *D*_ο_ is the diffusion coefficient (cm^2^ s^–1^), *C* is the analyte concentration (mol cm^–3^), and ν is the scan rate (V s^–1^).^12,30,51^

However, relying solely on the *I*_p_ *vs* *ν* ^1/2^ relationship or the minimal peak potential shift observed in Figure S5a (+0.642 to +0.661 V) can be insufficient for fully characterizing irreversible systems where surface processes may significantly influence the kinetics. Therefore, a more detailed kinetic analysis was performed. The relationship between log *I*_p_ and log ν (Figure S6a) yields a linear plot described by the equation log *I*_p_ (A) = 0.921 (log ν) – 5.575 (*R* ^2^ = 0.995). Importantly, the slope of 0.921 is significantly closer to the theoretical value of 1.0 expected for an adsorption-controlled process than to the value of 0.5 expected for pure diffusion control. Furthermore, a plot of *I*_p_ *vs* *ν* (Figure S6b) also exhibits strong linearity (*R* ^2^ = 0.992), following the equation *I*_p_ (A) = 3.032 × 10^–6^ (ν) + 4.421 × 10^–10^, which further supports a mechanism where the rate-determining step is linked to surface adsorption.^30,52^ This combined analysis provides stronger evidence that while diffusion contributes to mass transport (leading to the linearity observed in Figure S5b), the electrochemical reaction rate under these conditions is predominantly governed by the adsorption of OTA onto the SPE CB-G electrode surface.^12,13,49^ Thus, the oxidation of OTA under these conditions exhibits mixed control, with predominant adsorptive behavior. These findings emphasize the importance of integrating multiple kinetic analyses to accurately elucidate the underlying electrochemical mechanisms.

#### 3.5.2 Effect of CTAB on OTA Oxidation and Detection in PB pH 7.0

The enhancement of the electrocatalytic oxidation signal for OTA observed in the presence of CTAB can be attributed to a synergistic interaction among the surfactant, the analyte, and the SPE CB-G surface. CTAB, a cationic surfactant, possesses a characteristic molecular structure composed of a hydrophobic tail (long alkyl chain) and a positively charged hydrophilic head (trimethylammonium group) (Figure S7a).^19,27,34,36,53^

The *in situ* formation of an organized CTAB monolayer on the SPE CB-G surface is essential for the electrocatalytic oxidation of OTA. The interaction mechanism begins with the adsorption of CTAB’s hydrophobic tails onto the predominantly hydrophobic carbon black and graphite components of the electrode surface, primarily through van der Waals forces and hydrophobic interactions.^36,54^ This adsorption, at concentrations below the critical micelle concentration (CMC) of CTAB (approximately 0.92 mmol L^–1^),^19,53^ leads to an organized surfactant layer where the positively charged head groups are oriented toward the aqueous PB buffer solution (pH 7.0), as illustrated in Figure S7b.^19,27,34,36,53^

This CTAB-modified interface creates an ordered and positively charged microenvironment. At the working pH of 7.0, OTA exists predominantly in its anionic forms (OTA^–^ and OTA^2–^, see Figures 4 and S4).^10,17,18^ These anionic OTA species are electrostatically attracted to and accumulate on the CTAB-modified electrode surface.^19^ This preconcentration effect was demonstrated by the significant increase in the anodic *I*_p_ for OTA oxidation when CTAB was present, especially under conditions maximizing OTA accumulation (deposition potential, time, and stirring rate), resulting in up to a 54-fold signal enhancement compared to the absence of CTAB (Figure S8a,b). This improvement highlights the role of CTAB in facilitating electron transfer and reducing the background noise.^19,27,34,36^

To further elucidate the role of CTAB in modulating the interfacial kinetics, EIS measurements were performed. The impedance diagrams in the complex plane obtained for OTA (25 μmol L^–1^) in 0.2 mol L^–1^ PB buffer (pH 7.0), in the absence and presence of CTAB (80 μmol L^–1^), are shown in Figure S9, along with the equivalent Randles circuit model used for fitting. A significant decrease in the *R*_*ct*_ was observed upon the addition of CTAB. The *R*_*ct*_ value decreased dramatically from approximately 80 kΩ in the absence of CTAB to about 3.6 kΩ in its presence—a reduction of over 95%. This pronounced decrease in *R*_*ct*_ provides direct evidence that the CTAB layer substantially facilitates the electron transfer kinetics for the OTA oxidation reaction at the electrode–solution interface under conditions where the Faradaic reaction is occurring. The enhanced proximity and preorientation of OTA molecules, mediated by the CTAB layer, are likely key factors contributing to this improved interfacial kinetic behavior.^55^

The electrochemical behavior of OTA on the CTAB-modified SPE CB-G was further investigated by studying the effect of scan rate (ν) on the voltammetric response. Cyclic voltammograms obtained at scan rates ranging from 25 to 350 mV s^–1^ are shown in Figure 5a. A linear relationship between *I*_p_ and ν is evident in Figure 5b (*r* = 0.991; *R* ^2^ = 0.982), confirming that the OTA oxidation process is predominantly adsorption-controlled under these conditions.^19,27,36,37^ The regression equation *I*_p_ (A) = 1.626 × 10^–4^ (ν) + 6.912 × 10^–6^ indicates a robust model and reproducible results.

**Figure 5:**
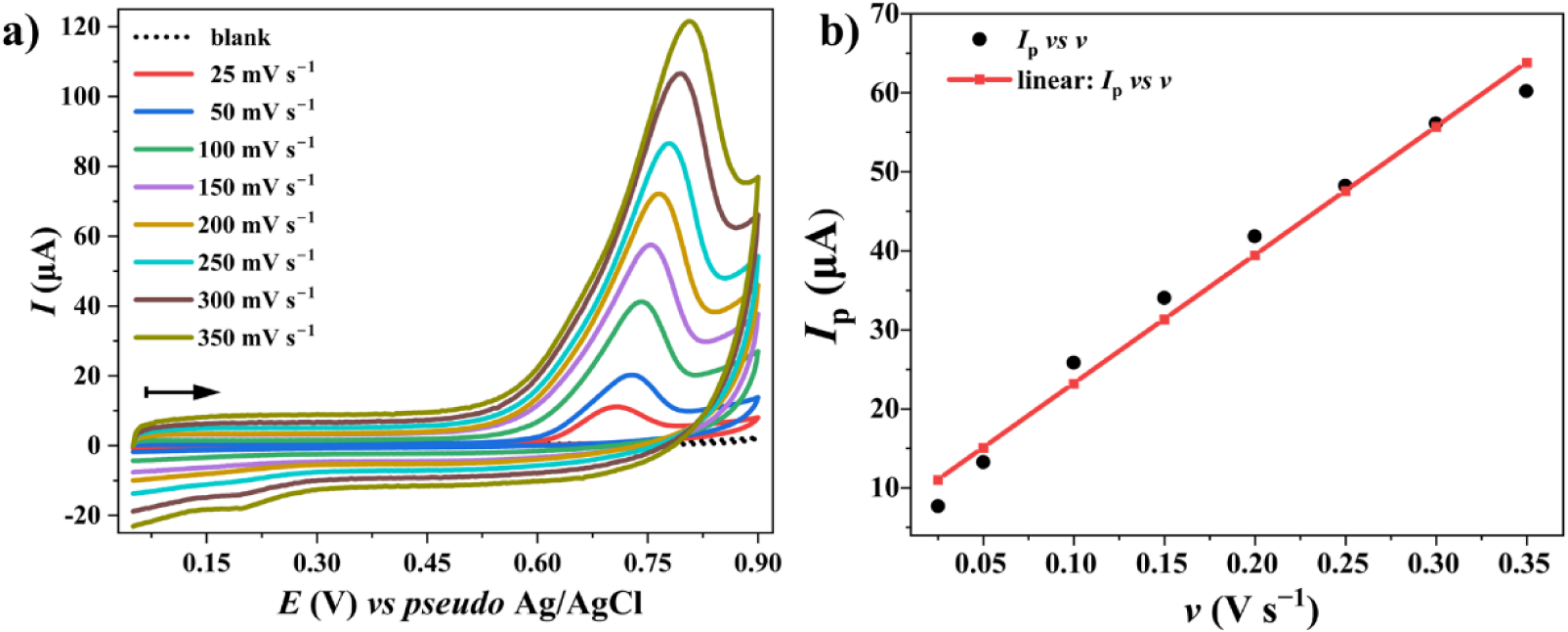
Cyclic voltammograms recorded at different scan rates (25–350 mV s^–1^) in 0.2 mol L^–1^ PB buffer (pH 7.0) containing CTAB (80 μmol L^–1^) and OTA (10 μmol L^–1^). The arrow indicates the direction of potential scanning. Deposition parameters: *E*_dep_ = +0.55 V, *t*_dep_ = 205 s, and stirring rate = 500 rpm. (b) Linear relationship between anodic *I*_p_ and ν, following the equation *I*_p_ (A) = 1.626 × 10^–4^ (ν) + 6.913 × 10^–6^ (*R* ^2^ = 0.982).

To further elucidate the OTA oxidation mechanism in the presence of CTAB, additional kinetic analyses were performed. The analysis of the log–log relationship between *I*_p_ and ν (Figure S10a) yielded a slope coefficient of 0.793 (*R* ^2^ = 0.996), which is close to the theoretical value of 1 characteristic of adsorption-controlled mechanisms.^30,53^ This finding supports the hypothesis that OTA oxidation occurs primarily through adsorption on the CTAB-modified electrode. Concurrently, the relationship between *I*_p_ and ν^1/2^ (Figure S10b) revealed a strong linear correlation (*R* ^2^ = 0.997), indicating that diffusion also contributes to the mass transport of OTA to the electrode surface. Taken together, these results suggest a mixed adsorption-diffusion control behavior for OTA oxidation on the CTAB-modified SPE, with adsorption playing a predominant role in defining the voltammetric response, especially under the preconcentration conditions employed.^19,27,34,53^

These findings are in agreement with previous adsorptive voltammetry studies, which showed that the presence of CTAB enhances OTA accumulation on the electrode surface, thereby improving the sensitivity of the method.^19,27^ The combination of the inherent properties of the SPE CB-G (high surface area provided by carbon black, excellent electrical conductivity of graphite) with CTAB-mediated analyte accumulation and facilitated interfacial electron transfer kinetics (as confirmed by EIS) results in the observed enhancement of the electrocatalytic oxidation signal, leading to a more sensitive and selective determination of OTA.^19,27,36,37,46,53,55–57^

### 3.6 Optimization of DPV and AdSDPV Parameters

DPV and AdSDPV were selected for OTA detection due to their high sensitivity, enhanced resolution, and effective suppression of capacitive current—key attributes for trace-level analysis in complex matrices.^12,28,30,51,58^ Instrumental parameters and solution concentrations for both DPV and AdSDPV were optimized using a CCD, enabling a systematic evaluation of variables to improve the analytical response while minimizing experimental effort.^38,40,41,59^

#### 3.6.1 DPV: Optimization and Effects

The optimization of the DPV parameters for OTA detection aimed to maximize the anodic *I*_p_ response. The CCD was employed to investigate the influence of *E*_pulse_ (mV) and *t*_pulse_ (ms). The voltammograms obtained from these experiments are presented in Figure S11, and the measured responses (*I*_p_, μA) in the presence of 5 μmol L^–1^ OTA in 0.2 mol L^–1^ PB at pH 7.0 are presented in Table S4.

The statistical analysis of the experimental results provided significant insights into the influence of these parameters on the sensor’s response. The coded coefficients (Table S5) derived from the response surface analysis indicated that both *E*_pulse_ and *t*_pulse_ significantly affected *I*_p_, with *p*-values below 0.05. *E*_pulse_ exhibited a positive effect (0.042), whereas *t*_pulse_ had a negative effect (−0.059) on the response (Figure S12a, main effects plot; Supporting Information). This suggests that increasing *E*_pulse_ enhances *I*_p_, whereas increasing *t*_pulse_ reduces *I*_p_. This finding was corroborated by the Pareto chart (Figure S12b), where *t*_pulse_ presented a highly standardized effect well above the critical value (2.365), indicating statistical significance at the 95% confidence level.

Analysis of variance (Anova) corroborated these findings, demonstrating high *F*-values for both parameters (124.91 for *E*_pulse_ and 249.37 for *t*_pulse_) with exceedingly low probabilities (*p* < 0.05) (Table S6). The quadratic model exhibited an exceptional fit, with an *R* ^2^ value of 0.986 and an adjusted *R* ^2^ of 0.976. This result indicates that the quadratic model accounted for more than 98% of the variability in the response, thus demonstrating its robustness and reliability.

The response surface and contour plots (Figure S13a, b, respectively, Supporting Information) provided a clear visual representation of the relationship between the optimized parameters and sensor response. Based on the results obtained from the CCD analysis, the optimal conditions were *E*_pulse_ of 100 mV and *t*_pulse_ of 25 ms. These settings produced the highest current response, closely aligned with the predictions of the modeled response surface, thus ensuring the reliability and accuracy of the optimization process as the foundation for proceeding with AdSDPV studies in the future.

#### 3.6.2 AdSDPV: Role Preconcentration and CTAB

AdSDPV was employed in this study to enhance the sensitivity of OTA detection by exploiting the preconcentration effect on the electrode surface. This technique integrates the principles of adsorptive stripping voltammetry (AdSV) with DPV, improving the analytical performance for trace-level analyte quantification.^19,27,30,42,51,58^ In this configuration, a fixed deposition potential (*E*_dep_ = +0.55 V)^19,27,42^ was applied to accumulate OTA onto the electrode surface before the DPV scan, effectively increasing its local concentration and amplifying the anodic *I*_p_.

To optimize the AdSDPV parameters, a CCD was applied using two independent variables: deposition time (*t*_dep_, s) and CTAB concentration (μmol L^–1^). The optimization experiments were performed at an OTA concentration of 201.91 ng mL^–1^ (0.5 μmol L^–1^) in 0.2 mol L^–1^ PB buffer (pH 7.0). CTAB, a cationic surfactant, was introduced to promote OTA adsorption onto the electrode via electrostatic and hydrophobic interactions. The voltammograms obtained from these experiments are presented in Figure S14, and the measured responses (*I*_p_, μA) in the presence of 201.91 ng mL^–1^ OTA in 0.2 mol L^–1^ PB at pH 7.0 with CTAB are listed in Table S7.

Statistical analysis of the CCD results revealed that *t*_dep_ had a strong and statistically significant positive effect on *I*_p_ (coefficient = 1.014, *p* < 0.0001), indicating that extended deposition times enhance analyte accumulation and detection sensitivity (Table S8). In contrast, the linear effect of CTAB concentration was not statistically significant (coefficient = −0.018, *p* > 0.05), but its quadratic term was significant (coefficient = −0.539, *p* < 0.02), as shown in Figure S15a. This indicates a nonlinear relationship between CTAB concentration and *I*_*p*_, with signal enhancement limited to an optimal concentration range and decreasing at both lower and higher concentrations.

These results suggest that while CTAB facilitates OTA adsorption, its excessive accumulation or dilution may reduce the availability of active sites or alter interfacial organization, thus compromising the electrochemical response. This interpretation is supported by the Pareto chart (Figure S15b), where the *t*_dep_ appears as the dominant factor, while CTAB concentration exhibits a secondary but relevant quadratic influence at the 95% confidence level. These observations were corroborated by Anova, which indicated a high *F*-value (37.20) and an extremely low probability (*p* < 0.0001) of *t*_dep_ (Table S9).

Based on the response surface and contour plots (Figure S16a, b, respectively, Supporting Information), a plateau-like region is observed near the central point of the experimental domain. Although maximum *I*_p_ was not uniquely associated with a single concentration, 80 μmol L^–1^ was chosen as the optimal CTAB value because (i) it corresponds to the central point of the CCD, ensuring minimal variation and high reproducibility, and (ii) it lies within the region of stable performance, avoiding suboptimal effects observed at the extremes of the tested range (24–137 μmol L^–1^). Importantly, this concentration remains well below the CMC of CTAB, thus minimizing the risk of micelle formation and preserving electrode accessibility levels.^19,27,30,42,51,53^ The model for AdSDPV demonstrated good predictive performance, with an *R* ^2^ of 0.871 and an adjusted *R* ^2^ of 0.778. These results underscore the robustness of the model and its ability to capture the effects of the optimized variables.

The optimized parameters—*t*_dep_ = 205 s and CTAB = 80 μmol L^–1^—were adopted for all subsequent analyses. Under these conditions, the sensor exhibited significantly enhanced anodic responses due to the synergistic effects of preconcentration and CTAB-mediated adsorption. As illustrated in Figure 6a, the AdSDPV configuration with CTAB produced an *I*_p_ approximately five times greater than DPV without CTAB. Figure 6b further confirms the superior performance of the optimized method in terms of signal intensity.

**Figure 6:**
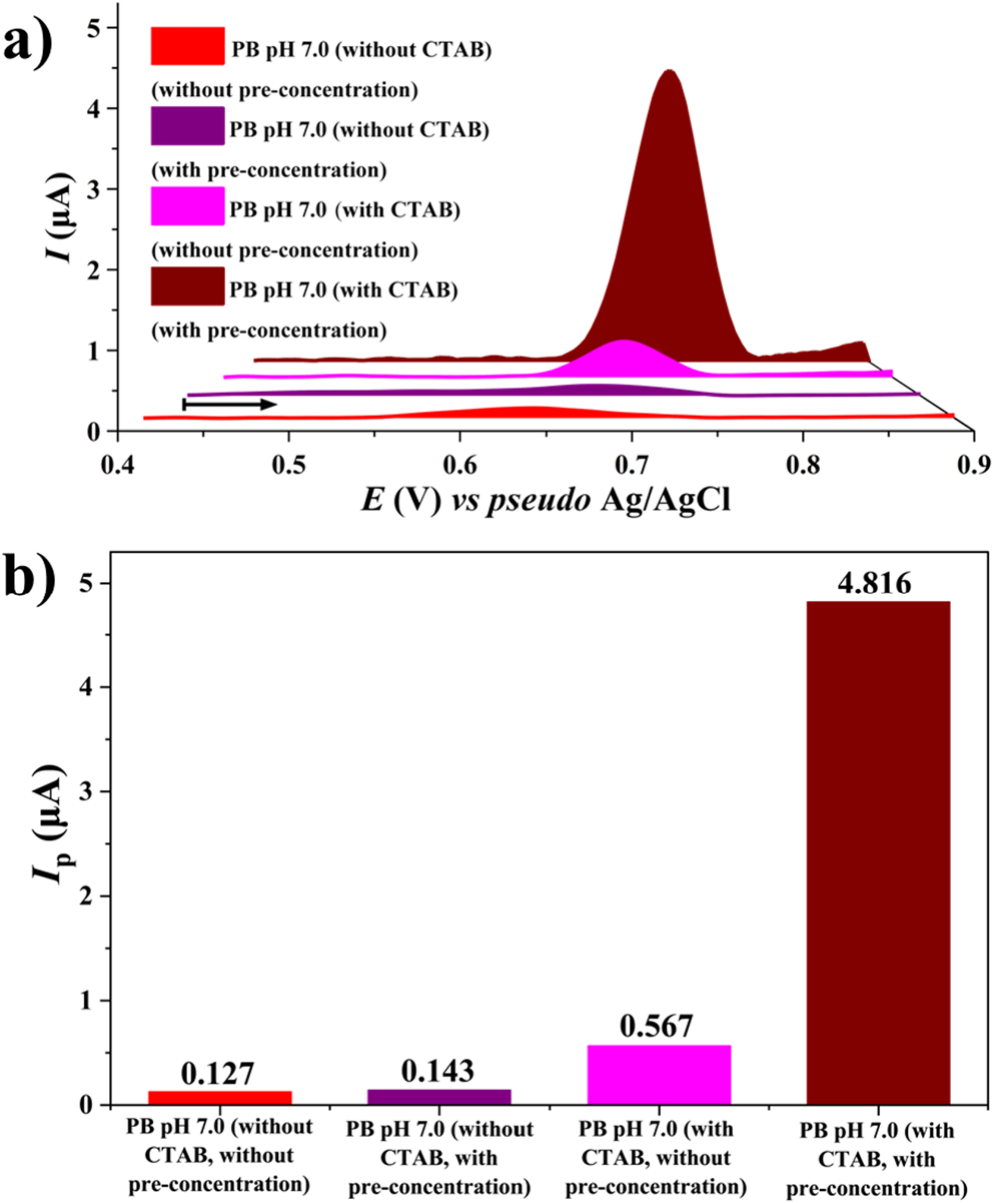
Differential pulse voltammograms (DPV), differential pulse anodic stripping voltammograms (DPASV), and adsorptive stripping differential pulse voltammograms (AdSDPVs) of OTA oxidation in 0.2 mol L^–1^ PB buffer pH 7.0 under four conditions: DPV without CTAB (red), DPASV without CTAB (purple), DPV with CTAB (pink), and AdSDPV with CTAB (dark red). OTA concentration: 201.91 ng mL^–1^ (0.5 μmol L^–1^); CTAB: 80 μmol L^–1^. The arrows indicate the scanning direction. The parameters of DPV, DPASV, and AdSDPV were *E*_pulse_ = 100 mV and *t*_pulse_ = 25 ms. Deposition parameters: *E*_dep_ = +0.55 V and *t*_dep_ = 205 s and stirring rate = 500 rpm. Scan rate: 10 mV s^–1^; step potential: 5 mV. (b) Comparison of the anodic *I*_p_ values for OTA oxidation under the same conditions as in Figure a.

These results provide a robust analytical foundation for the quantification of OTA at trace levels and validate the combined approach of statistical optimization with *in situ* modification of commercial electrochemical sensors. This strategy proved effective in enhancing analytical performance without the need for developing new devices, thereby simplifying its application in real-sample analysis.

### 3.7 Calibration Curve and Analytical Performance

The voltammetric determination of OTA was performed under optimized conditions using AdSDPV. The resulting voltammograms (Figure 7a) exhibited well-defined oxidation peaks, with anodic *E*_p_ ranging from +0.73 to +0.75 V (*vs pseudo* Ag/AgCl), and relative standard deviation (RSD) values between 0.40 and 1.0%. The anodic *I*_p_ displayed a linear correlation with OTA concentrations within the range of 10.10 to 242.29 ng mL^–1^ in 0.2 mol L^–1^ PB buffer (pH 7.0) containing 80 μmol L^–1^ of CTAB. However, for OTA concentrations between 242.29 and 403.81 ng mL^–1^, the *I*_p_ values deviated from linearity, likely due to the saturation of adsorption sites on the electrode surface. At these higher analyte concentrations, the limited number of active sites may become fully occupied, restricting further accumulation of OTA and leading to a plateau or diminished current response. Triplicate measurements (*n* = 3) resulted in RSD values ranging from 6.0% to 12.6%. The calibration curve (Figure 7b) exhibited excellent linearity, as described by the regression equation *I*_p_ (μA) = 0.0238­[OTA] – 0.286, *R* ^2^ = 0.994, and *r* = 0.997. The Anova of the calibration curve (Table S10) confirmed the statistical significance of the linear model (*F* = 972.31, *p* < 0.05) with a mean squared error of 0.030 (six degrees of freedom), thus validating the model for OTA quantification with a 95% confidence level.

**Figure 7:**
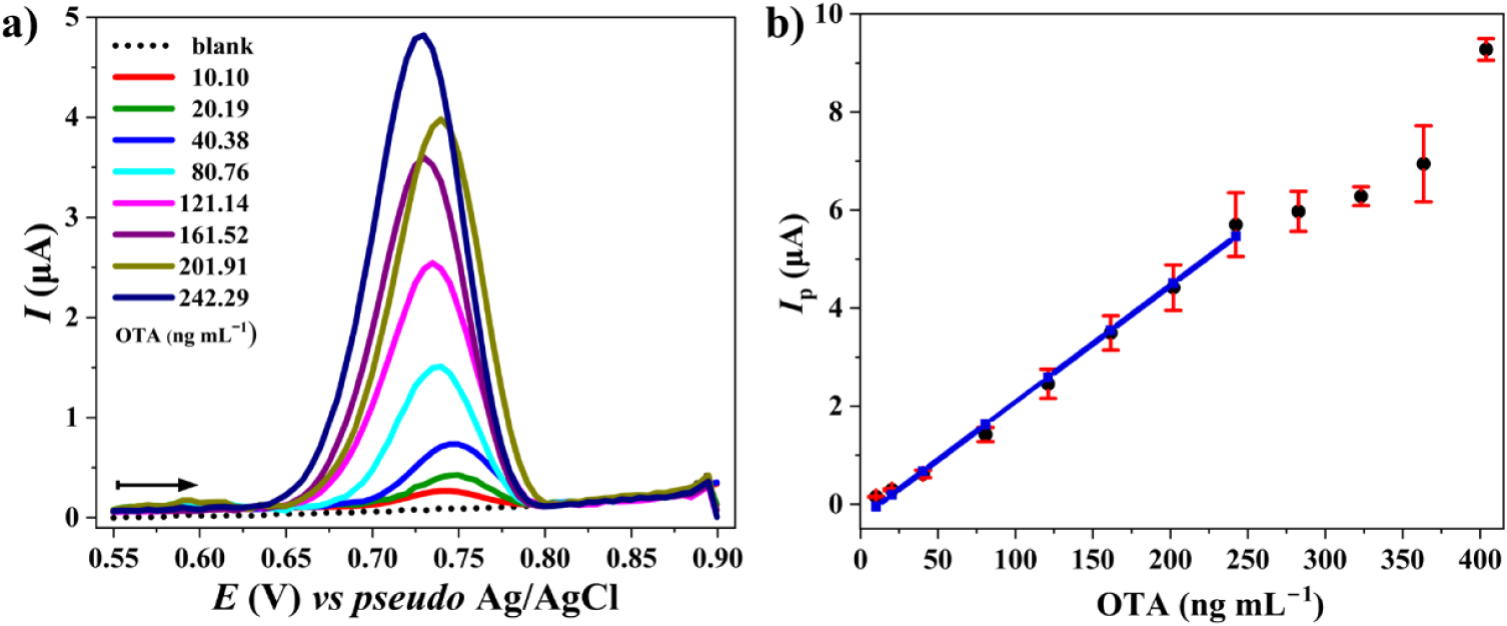
Adsorptive stripping differential pulse voltammograms (AdSDPV) obtained for SPE CB-G in the presence of increasing concentrations of OTA (10.10, 20.19, 40.38, 80.76, 121.14, 161.52, 201.91, and 242.29 ng mL^–1^) in 0.2 mol L^–1^ PB buffer (pH 7.0) containing CTAB (80 μmol L^–1^). The arrows indicate the scanning direction. The AdSDPV parameters were *E*_pulse_ = 100 mV, *t*_pulse_ = 25 ms, *E*_dep_ = +0.55 V, *t*_dep_ = 205 s, and stirring rate = 500 rpm. Scan rate: 10 mV s^–1^; step potential: 5 mV. (b) Calibration curve (*I*_p_ (μA) = 0.0238­[OTA] – 0.286; *R* ^2^ = 0.994; *n* = 3). Error bars represent standard deviation.

The LOD and limit of quantification (LOQ) were calculated using the standard deviation of the lowest OTA concentration (σ = 0.010) and the slope of the calibration curve (*S* = 0.0238 μA ng^–1^ mL^–1^), in accordance with the following equations: LOD = 3.3σ/*S* = 1.39 ng mL^–1^ and LOQ = 10σ/*S* = 4.20 ng mL^–1^. The approach for determining the LOD and LOQ was consistent with the guidelines provided by international and national standards, utilizing the standard deviation of the lowest analyte concentration. This approach is especially well-suited for instrumental techniques such as voltammetry, in which the standard deviation of the response is influenced by either the signal noise of the blank or the standard deviation of the lowest concentration of the analyte.^12,28,60–62^

The precision of the method was assessed by repeatability and reproducibility studies (Figure 8) using an OTA concentration of 201.91 ng mL^–1^. Repeatability was evaluated by performing three consecutive measurements on the same SPE, whereas reproducibility was assessed using nine different SPEs (S01–S09), resulting in 27 measurements. In addition, these assays were conducted on different days using different SPE connectors and standard OTA solutions prepared in 0.2 mol L^–1^ PB buffer (pH 7.0) containing 80 μmol L^–1^ of CTAB on the respective days of the electroanalytical experiments.

**Figure 8:**
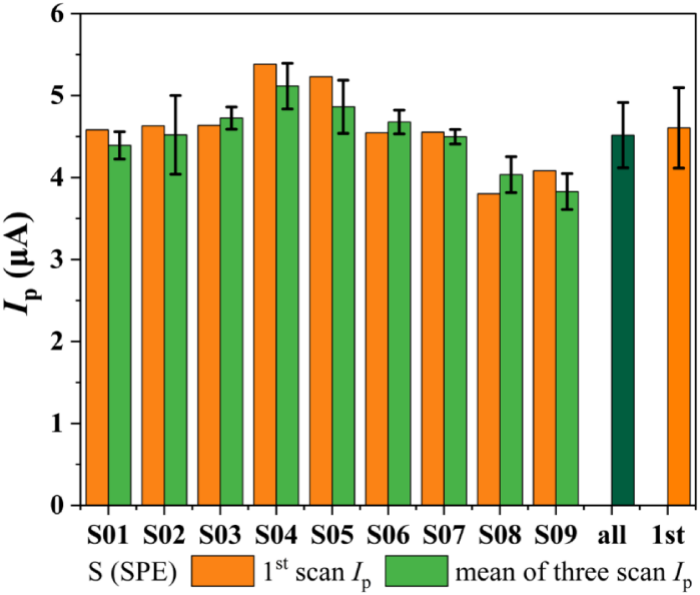
Evaluation of repeatability of the electrochemical method for OTA (201.91 ng mL^–1^) using AdSDPV. Bars represent: orange (S01–S09): *I*_p_ from the first scan on individual SPEs. Light green (S01–S09): mean *I*_p_ from three consecutive scans on individual SPEs (intraelectrode repeatability). Dark green (“all”): overall mean *I*_p_ from all measurements (3 scans/SPE × 9 SPEs = 27 total), assessing interelectrode reproducibility. Orange (“first”): mean *I*_p_ of only the first scan across all nine SPEs. Error bars denote standard deviation.

The results indicated an overall mean anodic *I*_p_ of 4.52 ± 0.40 μA across the 27 assays, with intraelectrode RSD values ranging from 1.97 to 10.62%, and an interelectrode RSD of 8.81%. The anodic *E*_p_ showed an overall mean of +0.74 ± 0.01 V (*vs pseudo* Ag/AgCl), with an interelectrode RSD of approximately 2%. These results demonstrate the robustness of the method, with acceptable variability between the different electrodes and consecutive measurements.

A comparison between the first measurement and the average of the three scans on the same electrode revealed satisfactory agreement, indicating good sensor stability during multiple analyses. However, performing a fourth scan on the same electrode significantly increased the intraelectrode RSD (RSD > 12%). Furthermore, traditional aqueous or electrochemical cleaning after surfactant use is not viable because it increases the background or residual current, negatively affecting the reproducibility and stability of subsequent readings. Thus, a new electrode was used for each OTA concentration measurement to ensure consistent and reliable results.^31,38,63^

To verify this method, recovery tests were conducted using 0.2 mol L^–1^ PB buffer (pH 7.0) and wheat extract (Table 2). The results showed good reproducibility across different SPEs, with RSD values ranging from 7.72 to 10.95%. In the PB buffer with CTAB, the OTA recovery rates ranged from 94.03 to 107.21%, demonstrating acceptable accuracy across all tested concentrations. In wheat extract, the OTA recovery was 109.50%, with an RSD of 8.25%, indicating good precision and satisfactory performance, even in a complex matrix. These results support the reliability of the method for OTA determination in both simple and complex matrices, with recovery values within the acceptable range for analytical methods (80–110%).^61,64^

**Table 2:** Analytical Performance Data for OTA Determination Using Different SPEs in 0.2 mol L^–1^ PB Buffer pH 7.0 with CTAB and Wheat Extract^a^ ^b^ ^c^

**OTA ng mL** ^–1^	Mean^a^ *I*_**p**_ **± SD (μA)**	**RSD** ^a^ **(%)**	**[OTA] found ± SD (ng mL**^**–1**^)	**RSD** ^b^ **(%)**	**Recovery (%)**
20.0	0.224 ± 0.049	21.99	21.44 ± 2.07	8.47	107.21
40.0	0.630 ± 0.071	11.21	38.49 ± 2.97	7.72	96.22
210.0	4.554 ± 0.517	11.35	203.36 ± 21.71	10.62	96.84
210.0	4.413 ± 0.515	11.66	197.45 ±21.62	10.95	94.03
100.0 (wheat)**	2.320 ± 0.214	9.26	109.51 ± 9.03	8.25	109.50

a Mean of *n* = 3 replicate measurements. For the PB buffer containing CTAB results, the mean *I*_p_ was obtained from three consecutive scans on the same SPE per replicate.

b For the wheat extract results, due to observed matrix effects inhibiting subsequent scans, a new SPE was used for each replicate measurement, and only the *I*_p_ from the first scan was recorded.

c The first RSD^a^ (%) refers to the relative standard deviation of the anodic peak current (*I*_p_, μA), while the second RSD^b^ (%) corresponds to the calculated OTA concentrations (ng mL^–1^) based on the calibration curve.

#### 3.7.1 Stability and Reusability Studies

Assessment of stability and reusability is essential for the practical application of electrochemical sensors. Therefore, detailed studies were conducted to evaluate the performance of SPE CB-G electrodes modified *in situ* with CTAB under repeated electrochemical analyses (AdSDPV) and subsequent reuse. These additional experiments were conducted using SPE CB-G electrodes from a new manufacturing batch.

The stability was evaluated over 30 consecutive AdSDPV scans for OTA (201.91 ng mL^–1^ in 0.2 mol L^–1^ PB buffer (pH 7.0) with 80 μmol L^–1^ CTAB) on new electrodes. For instance, with SPE 01, the *I*_p_ decreased by approximately 8.9% after 5 scans, 20.6% after 10 scans, and up to 58.0% after 30 scans relative to the first measurement. An electrode SPE 02 showed an initial phase of slight signal increase (+4.7% after 5 scans, indicating conditioning), followed by a more gradual decay, with a signal drop of 30.1% after 30 scans (detailed data in Table S11). This indicates that while the electrode supports several measurements, prolonged continuous use leads to significant signal attenuation, likely due to the adsorption of reaction products and/or surface fouling phenomena.

The reusability of the SPEs was also assessed. After an initial set of 30 scans, electrodes were gently rinsed with ultrapure water and stored under protective conditions. SPE 01 exhibited a current response 28.1% lower upon interday reuse (next day) compared to its first measurement when new. Intraday reuse of SPE 02 (on the same day, after 30 initial scans and rinsing) resulted in an initial current 21.4% lower than its first scan when new. In both reuse scenarios, the signal continued to degrade over subsequent scans during reuse.

Furthermore, the impact of use/reuse on electrode characteristics was examined by comparing blank voltammograms (PB buffer pH 7.0 + CTAB without OTA). Cyclic voltammetry analyses before and after the AdSDPV experiments (Figure S17a) demonstrated an elevation of the capacitive current after reuse for both SPE 01 and 02. This is attributed to the accumulation of substances and reaction products on the electroactive surface after initial use, potentially leading to increased electrical resistance, a decrease in the efficiently available area for electron transfer, and irreversible changes in the electrode’s surface properties. These observations are reinforced by background AdSDPV voltammograms (without OTA), which showed a marked increase in baseline currents and slope after reuse (Figure S17b). A direct consequence is the deterioration of the analytical response.^30,58^

These findings confirm that while the SPE CB-G electrodes modified in situ with CTAB offer good performance in initial measurements, their characteristics change significantly with repeated use or after exposure to OTA and its oxidation products in the presence of CTAB. This reinforces the single-use protocol for each replicate, especially to ensure accuracy in trace analyses by minimizing issues such as fouling, memory effects, and signal degradation. The interelectrode reproducibility (RSD 8.81%, Figure 8) with new SPEs thus remains the most relevant metric for the robustness of the method in practical applications.

### 3.8 OTA Detection in Coffee: Calibration and Matrix Interferences

The analysis of the matrix effects and compounds present in coffee using the electroanalytical method developed for OTA determination provided relevant insights into the selectivity of the method. Additionally, this study highlights the applicability of this method to complex matrices. The same parameters used for OTA detection in 0.2 mol L^–1^ PB buffer at pH 7.0 with 80 μmol L^–1^ CTAB were applied to the electrochemical analysis of OTA in coffee extract using SPE CB-G added to the 0.2 mol L^–1^ PB buffer (pH 7.0) with 80 μmol L^–1^ CTAB.

The voltammograms (Figure 9a) revealed well-defined anodic *E*_p_ between +0.73 and +0.75 V (*vs pseudo* Ag/AgCl), with an RSD ranging from 1.0 to 2.0%. Anodic *I*_p_ exhibited a strong linear relationship with OTA concentrations ranging from 10.0 to 210.0 ng mL^–1^ in the coffee extract using 0.2 mol L^–1^ PB buffer (pH 7.0) with 80 μmol L^–1^ CTAB. However, the AdSDPV voltammograms (Figure S18) revealed a larger peak width at half-height (*W*_1/2_ = 0.090 V) for OTA in the coffee matrix compared with the OTA standard (*W*_1/2_ = 0.070 V), indicating matrix effects from the compounds present in the matrix on the electron transfer processes at the electrode surface.

**Figure 9:**
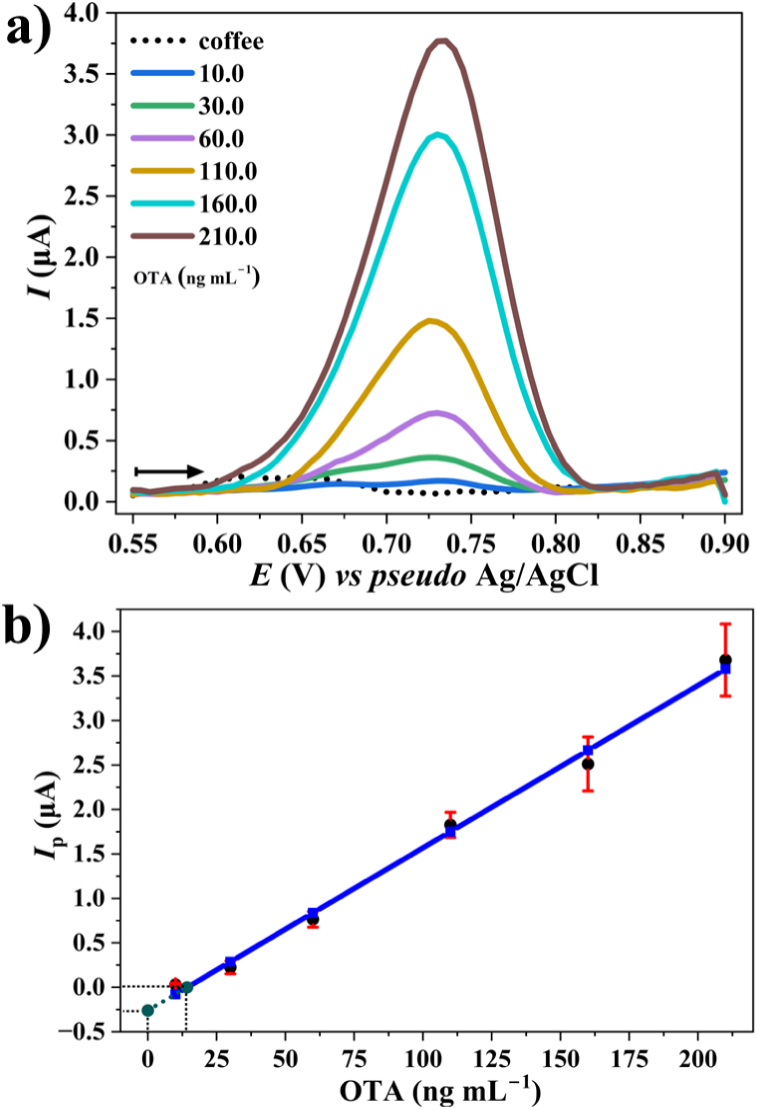
Adsorptive stripping differential pulse voltammograms (AdSDPV) obtained for SPE CB-G in the presence of increasing concentrations of OTA in coffee extract (10.0, 30.0, 60.0, 110.0, 160.0, and 210.0 ng mL^–1^) in 0.2 mol L^–1^ PB buffer (pH 7.0) modified with CTAB (80 μmol L^–1^). The arrows indicate the scanning direction. The AdSDPV parameters were *E*_pulse_ = 100 mV, *t*_pulse_ = 25 ms, *E*_dep_ = +0.55 V, *t*_dep_ = 205 s, and stirring rate = 500 rpm. Scan rate: 10 mV s^–1^; step potential: 5 mV. (b) Calibration curve (*I*_p_ (μA) = 0.0183­[OTA] – 0.260; *R* ^2^ = 0.994; *n* = 3). Error bars represent standard deviation.

Measurements for each concentration were performed in triplicate (*n* = 3), with RSD values decreasing from 45.0% at 10.0 ng mL^–1^ to 8.0% at 110.0 ng mL^–1^. The calibration curve (Figure 9b) exhibited excellent linearity, with the following regression equation: *I*_p_ (μA) = 0.0183­[OTA] – 0.260, *R* ^2^ = 0.994, and *r* = 0.997. The Anova of the calibration curve (Table S12) confirmed the adequacy and significance of the linear model (*p* < 0.05), with a mean square error of 0.015, indicating acceptable variability around the regression line.

The sensitivity of the method was evaluated by calculating the LOD and LOQ using the standard deviation of the lowest OTA concentration (σ = 0.015) and the slope of the calibration curve (*S* = 0.0183 μA^–1^ ng mL^–1^). The resulting values, LOD = 3.3σ/*S* = 2.70 ng mL^–1^ and LOQ = 10σ/*S* = 8.20 ng mL^–1^, demonstrate the high sensitivity of the method.^61,62,64^ The observed increase in the OTA oxidation signal in the presence of CTAB highlights its role in facilitating electron transfer processes, even in complex matrices such as coffee.^19,27,36,56,65,66^

#### 3.8.1 Recovery and Precision in the Coffee Matrix

The analysis of OTA recovery in the coffee matrix revealed satisfactory results across all evaluated concentrations, ranging from 94.64% at 50 ng mL^–1^ to 109.86% at 180 ng mL^–1^ (Table 3). The RSD values remained below 14%, with better precision at higher concentrations. Variations in recovery could be attributed to the interactions between OTA and coffee compounds or adsorption/oxidation limitations at the electrode surface. The addition of CTAB was necessary to mitigate matrix interference and amplify the electrochemical signals of OTA.

**Table 3:** Summary of the Recovery and Precision of OTA Detection in Coffee Matrices at Three Different Concentrations^a^ ^b^ ^c^

**OTA added (ng mL**^**–1**^)	Mean^a^ *I*_**p**_ **± SD (μA)**	**RSD** ^a^ **(%)**	**OTA found ± SD (ng mL**^**–1**^)	**RSD** ^b^ **(%)**	**Recovery (%)**
50.0	0.606 ± 0.122	20.05	47.32 ± 6.64	14.03	94.64
120.0	1.854 ± 0.239	12.89	115.52 ± 13.07	11.31	96.27
180.0	3.359 ± 0.338	10.06	197.74 ± 18.45	9.33	109.86

a The recovery percentage demonstrated the robustness of the method for different OTA concentrations.

b Mean of *n* = 3 replicate measurements. Due to significant matrix interference from coffee inhibiting subsequent scans, a new SPE was used for each replicate measurement, and only the *I*_p_ from the first scan was recorded.

c The first RSD^a^ (%) refers to the relative standard deviation of the anodic peak current (*I*_p_, μA), while the second RSD^b^ (%) corresponds to the calculated OTA concentrations (ng mL^–1^) based on the calibration curve.

#### 3.8.2 Effect of Typical Coffee Interferents

The influence of typical coffee interferents and matrix effects on OTA determination was thoroughly investigated (Figure 10). The concentrations of the interferents, detailed in Table S3, were estimated based on typical literature^43–45^ values for coffee composition, adjusted for 1:50 sample dilution employed in the electrochemical measurement, as described in Section 2.6. Caffeine increased *I*_p_ by 19.67%, suggesting possible electrochemical synergy, although it was not electroactive in PB buffer at pH 7.0.^67,68^ Trigonelline and furfural exhibited negligible interference, with *I*_p_ reductions of 2.35 and 3.11%, respectively, indicating low competition with OTA.^69,70^ Acrylamide caused a 13.76% increase in *I*_p_,^71^ potentially influencing the sensor response through surface interactions or double-layer perturbations.

**Figure 10:**
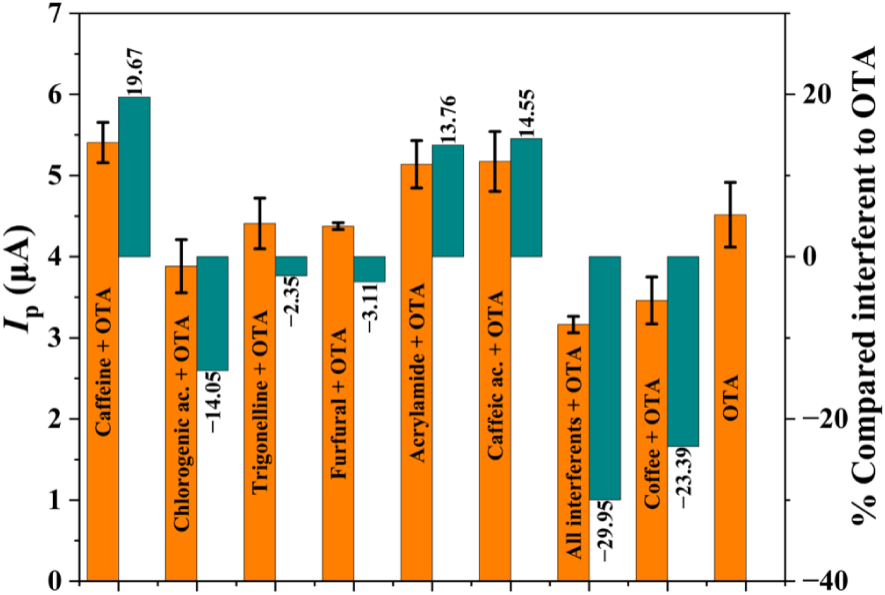
Influence of typical coffee interferents on *I*_p_ of OTA oxidation (201.91 ng mL^–1^) in 0.2 mol L^–1^ PB buffer (pH 7.0) modified with CTAB (80 μmol L^–1^) using SPE CB-G and AdSDPV. *I*_p_ values are represented by orange bars, and the percentage of variation relative to the standard OTA is indicated by green bars. Error bars represent standard deviation.

The presence of caffeic acid, a common phenolic compound in coffee, significantly enhanced the analytical signal by 14.55%. While its oxidation potentials (+0.20 to +0.65 V)^65,72^ are significantly lower than that of OTA (∼+0.74 V), precluding direct peak overlap, it likely competes with OTA for active sites modified by CTAB. Conversely, chlorogenic acid, another major phenolic constituent, resulted in a 14.05% reduction in *I*_p_. Its oxidation occurs at significantly lower potentials (+0.27 to +0.31 V) compared to OTA.^12,36,56,66,73–75^

For both phenolic interferents, the observed effects, despite occurring at potentials distinct from OTA’s oxidation, are attributed primarily to competitive adsorption onto the CTAB-modified surface. The electrochemical oxidation of such phenolics can yield quinone-like species; these products, or the parent phenolic compounds themselves, may adsorb and block active sites required for OTA accumulation and subsequent oxidation, rather than causing direct signal interference at the OTA peak potential. This highlights the complexity of the matrix effects and the importance of electrode surface modifications.^12,36,56,66,73–75^

Collectively, the interferents resulted in an approximately 30% reduction in the electrochemical signal of OTA. The coffee matrix alone, even when diluted (100 μL extract in 5 mL of 0.2 mol L^–1^ PB buffer at pH 7.0 with 80 μmol L^–1^ CTAB), caused a 23.4% decrease in *I*_p_ value. These findings underscore the complexity of analyte-matrix interactions.^14^ However, the addition of CTAB was critical for minimizing the matrix effects and interference. This enabled SPE CB-G to achieve high selectivity and reliable performance, even at low OTA concentrations.

#### 3.8.3 Comparison with Other Electrochemical Sensors from the Literature

The analytical performance of the developed method was evaluated and compared with that of various electrochemical sensors reported in the literature (Table 4). These comparisons highlight the practicality, sensitivity, and potential applicability of the proposed method for detecting OTA in real-world matrices.

**Table 4:** Comparison of the Developed Electrochemical Sensor with Other Sensors Reported in the Literature for OTA Detection

Technique/Electrode^c^ ^b^	**Sample**	Buffer^a^	**LOD/LOQ**	**Linear range**	**ref.**
		mol L^–1^	ng mL^–1^		
DPV (anti-OTA/DIAZO/SPCE)	Corn, wheat, rice, coffee beans, and wine	PB 0.1 pH 7.4	0.50/–	20.0–200.0	^76^
DPV (CB-G-CPE)	Durum wheat	BR 0.04 pH 5.0	23/77	11.20–2220.0	^28^
EIS (Au/Cys/anti-OTA)	Roasted coffee	PB 0.01 pH 7.2	0.15/–	0.50–100.0	^29^
AdSDPV (CNTP + zephiramine)	–	PB 0.1 pH 7.2	444.20/–	2019.0–20,190.0	^19^
DPV (CTAB-Au(III)/GCE)	Beer	PBS 0.1 pH 7.0	8.08/–	40.38–4038.0	^27^
DPASV (GNP/CPE)	Baby food, breakfast cereals, and beer	PBS 0.1 pH 7.2	0.08/–	0.20–40.38	^42^
CA SPGE-oleamide	Cow’s and vegetarian milk	PB 0.1 pH 7.4	–/0.0004	0.0004–40.38	^77^
AdSDPV (SPE CB-G + CTAB)	Wheat	PB 0.2 pH 7.0	1.39/4.20	10.09–242.29	This work
AdSDPV (SPE CB-G + CTAB)	Roasted coffee	PB 0.2 pH 7.0	2.70/8.20	10.0–210.0	This work

a PB: phosphate buffer; PBS: phosphate-buffered saline; BR: Britton–Robinson buffer.

b Anti-OTA/DIAZO/SPCE: screen-printed carbon electrode modified with diazonium salt and OTA antibodies; CB-G-CPE: graphite and carbon black as carbon paste electrode; Au/Cys/Anti-OTA: gold modified with cysteamine and OTA antibodies; CNTP + zephiramine: carbon nanotube paste modified with zephiramine; CTAB-Au­(III)/GCE: glassy carbon electrode modified with gold and CTAB; GNP/CPE: gold nanoparticles modified with carbon paste electrode; SPGE-oleamide: screen-printed gold electrode modified with (Z)-*N*-[2-(4-hydroxyphenyl)­ethyl]­octadec-9-enamide; SPE CB-G + CTAB: screen-printed carbon black and graphite electrode modified with CTAB.

c EIS: electrochemical impedance spectroscopy; DPV: differential pulse voltammetry; DPASV: differential pulse anodic stripping voltammetry, AdSDPV: adsorptive stripping differential pulse voltammetry; CA: chronoamperometry.

The use of SPE CB-G in combination with CTAB and AdSDPV demonstrated excellent analytical performance for OTA detection. Compared with other electrochemical sensors, our method offers a sensitivity that is comparable to or exceeds that of recent approaches. A sensor based on Au/Cys/anti-OTA using EIS achieved an LOD of 0.15 ng mL^–1^ in roasted coffee.^29^ Carbon paste electrodes modified with gold nanoparticles (LOD = 0.08 ng mL^–1^)^42^ and anti-OTA/DIAZO/SPCE sensors (LOD = 0.5 ng mL^–1^)^76^ showed good sensitivity for application in different types of food matrices. However, the use of antibodies requires precise immobilization techniques and stringent storage conditions, which limit the scalability and practicality of routine or field applications. The preparation of AuNPs increases fabrication costs, restricting their feasibility for routine analysis.

Other reported electrochemical approaches include the use of a carbon nanotube paste electrode combined with the surfactant zephiramine, achieving an LOD of 444.20 ng mL^–1^.^19^ Although economically viable, this method has a significantly lower sensitivity, making it less suitable for compliance with international regulations. A carbon black-graphite paste electrode (CB-G-CPE) for OTA analysis in durum wheat achieved a LOD of 23 ng mL^–1^,^28^ and a gold and CTAB-modified GCE for beer exhibited an LOD of 8.08 ng mL^–1^.^27^

More recently, a smartphone-operated stochastic sensor based on a screen-printed gold electrode (SPGE) modified with an oleamide derivative ((Z)-N-[2-(4-hydroxyphenyl)­ethyl]­octadec-9-enamide) has been proposed for the detection of OTA in cow’s milk and vegetarian milk.^77^ This system achieved an exceptionally low LOQ of 0.0004 ng mL^–1^. While this approach demonstrates outstanding sensitivity and is well-suited for point-of-need testing, it relies on gold electrodes and specialized modifiers, which may limit its scalability due to higher costs and fabrication complexity. In contrast, our CTAB-modified SPE CB-G platform, employing voltammetric detection via AdSDPV, offers a practical and cost-effective solution with sufficient sensitivity to meet international regulatory limits. Its ease of use, rapid analysis time, and compatibility with disposable carbon-based electrodes further support its applicability for decentralized and routine food safety monitoring.

The proposed method using SPE CB-G with CTAB strikes an optimal balance between sensitivity (LOD = 1.39 ng mL^–1^ in PB buffer and 2.70 ng mL^–1^ in coffee) and operational feasibility, enabling rapid and low-cost analysis. Unlike approaches involving antibodies or noble metals, our method avoids complex immobilization steps and costly materials, offering broader accessibility for laboratories equipped with standard voltammetric instrumentation.

Furthermore, the analytical method complies with the maximum OTA levels established by key regulatory agencies, including Anvisa (10 μg kg^–1^ for processed cereals and roasted coffee),^8^ the European Union (5.0 μg kg^–1^ for unprocessed cereals),^9^ and the recommended values for animal feed (250 μg kg^–1^).^78^ These results underscore the method’s reliability, regulatory alignment, and suitability for practical applications in food quality control.

## 4 Conclusion

This study successfully established a robust and cost-effective electrochemical sensing strategy for the detection of ochratoxin A (OTA) in complex food matrices such as coffee and wheat. By synergistically combining commercially available screen-printed electrodes (SPE CB-G) with *in situ* modification using the cationic surfactant CTAB and optimized adsorptive stripping differential pulse voltammetry (AdSDPV), the method achieved high sensitivity and selectivity while mitigating matrix interferences. The critical role of CTAB in enhancing OTA accumulation at the electrode interface and facilitating electron transfer kinetics was demonstrated, underscoring the value of surfactant-mediated approaches for improving sensor performance in challenging samples such as roasted coffee.

The developed method offers significant practical advantages for food safety monitoring, including operational simplicity, rapid analysis time, and compatibility with disposable, low-cost electrodes. Although each measurement requires a fresh electrode to ensure reproducibility and prevent surface fouling, the affordability of SPEs makes this approach practical for routine screening. The achieved detection limits comply with stringent regulatory standards for OTA, and validation studies in wheat and coffee confirmed satisfactory recovery rates, reinforcing the method’s reliability for real-world applications. This positions the sensor and the developed method as a promising alternative to conventional chromatographic techniques, particularly for decentralized or high-throughput quality control settings.

To further advance the practical impact and capabilities of this sensing platform, future studies could focus on validating the method across a broader range of food and feed matrices, such as spices, other cereals, nuts, and wine, thereby broadening its applicability. Additionally, exploring innovative electrode modifications, including alternative surfactants (zwitterionic or other cationic surfactants) or hybrid approaches integrating nanomaterials (carbon nanotubes, graphene, metallic nanoparticles) and molecularly imprinted polymers (MIPs), might enhance sensor selectivity, lower detection limits, and improve electrode reusability. The integration of the sensing platform into portable, user-friendly, or paper-based devices could further facilitate on-site testing, particularly beneficial in resource-limited settings. Finally, adapting the current approach to enable simultaneous detection of multiple mycotoxins or organic contaminants through tailored surfactant–analyte interactions or array-based sensor designs represents a promising future research direction.

In summary, this work not only delivers a practical tool for OTA monitoring but also contributes to the broader field of electrochemical sensing by demonstrating how surfactant-mediated strategies can effectively overcome matrix interferences. By bridging affordability with analytical rigor, this methodology paves the way for scalable, next-generation sensors to safeguard food supply chains.

## Supplementary Material



## Data Availability

All data supporting the findings of this study are available within the article and its Supporting Information.

## Notes

Detailed experimental designs, model results, statistical analyses (Anova), and stability studies (Tables S1–S12); electrode surface characterizations, OTA electrochemical behaviors, optimization outcomes, and sensor stability (Figures S1–S18) (PDF)
