# Supplementary material for: Optimized Electrochemical Sensor for Ochratoxin A Quantification in Coffee and Wheat Matrices
Source: ACS Omega. 2025 Jun 26;10(26):28252–68. doi: 10.1021/acsomega.5c03171 (PMC12242640; doi:10.1021/acsomega.5c03171)
Supplement: Supplementary file 1 [file ao5c03171_si_001.pdf]

Supporting Information

Optimized Electrochemical Sensor for Ochratoxin A quantification in Coffee and Wheat Matrices

Jane S. Medeiros[a], Jairo P. Oliveira[b], Helder Knidel[c], Tárcila M. N. Silva[a], José G. A. Rodrigues[a], Rafael Q. Ferreira[a*]

[a]Department of Chemistry/Center of Exact Sciences, Federal University of Espírito Santo, Fernando Ferrari Avenue, n° 514, 29075-910 Goiabeiras, Vitória – ES, Brazil

[b]Department of Morphology/Health Sciences Center, Federal University of Espírito Santo, Marechal Campos Avenue, nº 1468, 29047-105 Maruípe, Vitória – ES, Brazil

[c]Kaffee Group – ES-146 Highway, km 6.1, – 29255-000 Santa Maria, Marechal Floriano – ES, Brazil

Corresponding author: *Rafael de Queiroz Ferreira, Department of Chemistry/Center of Exact Sciences, Federal University of Espírito Santo, Fernando Ferrari Avenue, n° 514, 29075-910 Goiabeiras, Vitória, ES, Brazil.

E-mail address: rafael.q.ferreira@ufes.br (Rafael de Q. Ferreira)

Supporting Information Tables:

Tables S1-S12: Detailed experimental designs, model results, statistical analyses (ANOVA), and stability studies performed to optimize electrochemical parameters for DPV and AdSDPV techniques.

Table S1: Lists the independent variables (pulse potential - E_{pulse}; pulse time - t_{pulse}) and their respective real and coded levels used in the Central Composite Design (CCD) for the optimization of the Differential Pulse Voltammetry (DPV) parameters.

Table S2: Outlines the independent variables (CTAB concentration; deposition time - t_{dep}) and their real and coded levels employed in the CCD for the optimization of Adsorptive Stripping Differential Pulse Voltammetry (AdSDPV) parameters.

Table S3: Details of the common coffee matrix interferents investigated, their final concentrations used in the electrochemical cell during the interference tests, and the proportion of these concentrations relative to the fixed ochratoxin A (OTA) concentration.

Table S4: Presents the experimental design matrix and the results (measured peak current - I_p and peak potential - E_p) for the 13 runs of the CCD performed to optimize DPV parameters (E_{pulse}, t_{pulse}).

Table S5: Shows the coded regression coefficients, standard errors, confidence intervals, *t*-values, and probabilities (*p*-values) for the quadratic model fitted to the DPV optimization CCD results.

Table S6: Details the Analysis of Variance (ANOVA) results for the quadratic regression model fitted to the DPV optimization data, evaluating the statistical significance of the model and its individual terms.

Table S7: Presents the experimental design matrix and the results (measured peak current - I_p and peak potential - E_p) for the 13 runs of the CCD performed to optimize AdSDPV parameters (CTAB concentration, t_{dep}).

Table S8: Shows the coded regression coefficients, standard errors, confidence intervals, *t*-values, and probabilities (*p*-values) for the quadratic model fitted to the AdSDPV optimization CCD results.

Table S9: Details the Analysis of Variance (ANOVA) results for the quadratic regression model fitted to the AdSDPV optimization data, evaluating the statistical significance of the model and its individual terms.

Table S10: Presents the Analysis of Variance (ANOVA) results evaluating the linearity and statistical significance of the calibration curve regression model for OTA determination in PB buffer (pH 7.0).

Table S11: Details Intra-electrode stability and reusability assessment of SPE CB-G electrodes for OTA detection, showing % variation in I_p over 30 scans and inter-day reuse.

Table S12: Presents the Analysis of Variance (ANOVA) results evaluating the linearity and statistical significance of the calibration curve regression model for OTA determination in the coffee matrix extract.

Supporting Information Figures:

Figures S1-S18 illustrate electrode surface characterizations, OTA electrochemical behaviors, optimization outcomes, and sensor stability.

Figure S1: Illustrates the Raman micrographs of SPE CB-G surfaces. (**a**) Unmodified electrode (without CTAB); (**b**) electrode modified with CTAB, with a 60 µm scale bar. The red cross marks the spectral acquisition region.

Figure S2: Illustrates the Raman spectra of the SPE CB-G surface before (black line) and after (red line) *in situ* modification with 80 µmol L^{-1} CTAB.

Figure S3: Illustrates the 3D AFM topographical images (20.0 µm × 20.0 µm) of SPE CB-G: (**a**) unmodified and (**b**) CTAB-modified surfaces, revealing reduced roughness post-CTAB adsorption.

Figure S4: Illustrates the chemical structures of ochratoxin A (OTA) and its different protonation states (neutral, monoanionic, dianionic) and hydrolyzed form (open lactone ring) as a function of pH in aqueous solutions, including relevant pK_a values.

Figure S5: Presents (**a**) cyclic voltammograms of OTA oxidation in PB buffer (pH 7.0) at varying scan rates (25–350 mV s^{-1}) and (**b**) the linear relationship between anodic I_p and the square root of scan rates ($v^{1/2}$), providing insights into the diffusion-controlled mechanism of OTA oxidation.

Figure S6: Presents (**a**) linear relationship between log I_p *vs* log v and (**b**) the linear relationship between I_p *vs* v, supporting mixed diffusion/adsorption kinetics.

Figure S7: Presents (**a**) the molecular structure of the cationic surfactant cetyltrimethylammonium bromide (CTAB) and (**b**) a schematic representation of CTAB monolayer formation on the electrode surface and its interaction with anionic forms of OTA at PB buffer (pH 7.0).

Figure S8: Comparison of (**a**) cyclic voltammograms and (**b**) corresponding peak currents for OTA oxidation under different conditions (with/without CTAB, with/without pre-concentration), highlighting the signal enhancement achieved by combining CTAB and a pre-concentration step.

Figure S9: Impedance diagram for SPE CB-G with and without CTAB, including Randles equivalent circuit for data fitting.

Figure S10: Presents (**a**) Linear relationship between log I_p and log v; (**b**) Linear relationship between I_p and $v^{1/2}$ for OTA oxidation in CTAB-modified systems.

Figure S11: Displays the overlaid Differential Pulse Voltammograms (DPVs) recorded during the 13 experimental runs of the Central Composite Design (CCD) used to optimize the DPV parameters.

Figure S12: Illustrates the statistical analysis of the DPV optimization: (**a**) main effects plots showing the influence of pulse potential (E_{pulse}) and pulse time (t_{pulse}) on peak current (I_p), and (**b**) a Pareto chart ranking the significance of these effects and their interactions.

Figure S13: Relationship between the DPV parameters and peak current (I_p) response: (**a**) 3D response surface plot and (**b**) corresponding 2D contour plot showing the effects of pulse potential (E_{pulse}) and pulse time (t_{pulse}).

Figure S14: Displays the overlaid Adsorptive Stripping Differential Pulse Voltammograms (AdSDPVs) recorded during the 13 experimental runs of the CCD used for optimizing the AdSDPV parameters.

Figure S15: Illustrates the statistical analysis of the AdSDPV optimization: (**a**) main effects plots showing the influence of CTAB concentration and deposition time (t_{dep}) on peak current (I_p), and (**b**) a Pareto chart ranking the significance of these effects and their interactions.

Figure S16: Relationship between AdSDPV parameters and peak current (I_p) response: (**a**) 3D response surface plot and (**b**) corresponding 2D contour plot showing the effects of CTAB concentration and deposition time (t_{dep}).

Figure S17: Displays (**a**) cyclic voltammograms of SPEs before/after 30 AdSDPV runs, showing increased capacitive current; (**b**) Blank AdSDPVs demonstrating elevated background post-reuse.

Figure S18: Comparison of AdSDPV responses for blank PB buffer, blank coffee extract, OTA standard in PB buffer (pH 7.0), and OTA in the coffee matrix, illustrating the effect of the coffee matrix on the OTA oxidation peak shape (specifically, peak broadening).

Table S1. Variables and real/coded levels were studied in the central composite design (CCD) to optimize the I_p for OTA oxidation using DPV.

**Parameters**	**− α**	**Lower Level**	**Central Level**	**Upper Level**	**+ α**
	**−1.414**	**−1**	**0**	**+1**	**+1.414**
Pulse potential. (E_{pulse}, mV) – X_1	40 (39.65)	50	75	100	110 (110.35)
Pulse time (t_{pulse}, ms) – X_2	15 (14.65)	25	50	75	85 (85.35)

Table S2. Variables and real/coded levels were studied in the CCD to optimize the I_p response to OTA oxidation using AdSDPV.

**Parameters**	**− α**	**Lower Level**	**Central Level**	**Upper Level**	**+ α**
	**−1.414**	**−1**	**0**	**+1**	**+1.414**
CTAB concentration (μmol L^{-1}) – X_1	24 (23.43)	40	80	120	137 (136.57)
Deposition time (t_{dep}, s) – X_2	35 (35.14)	60	120	180	205 (204.86)

Table S3. Final concentrations and proportions of coffee matrix interferents relative to OTA in the electrochemical cells. The OTA concentration was fixed at 201.91 ng mL^{-1} to evaluate the effect of these interferents.

**Interferent**	**Final concentration (ng mL^{-1})**	**Proportion relative to OTA (%)**
Caffeine	19,500.0	96.58
Chlorogenic acid	17,600.0	87.17
Furfural	1,800.0	8.92
Trigonelline	4,200.0	20.81
Caffeic acid	900.0	4.46
Acrylamide	100.0	0.50

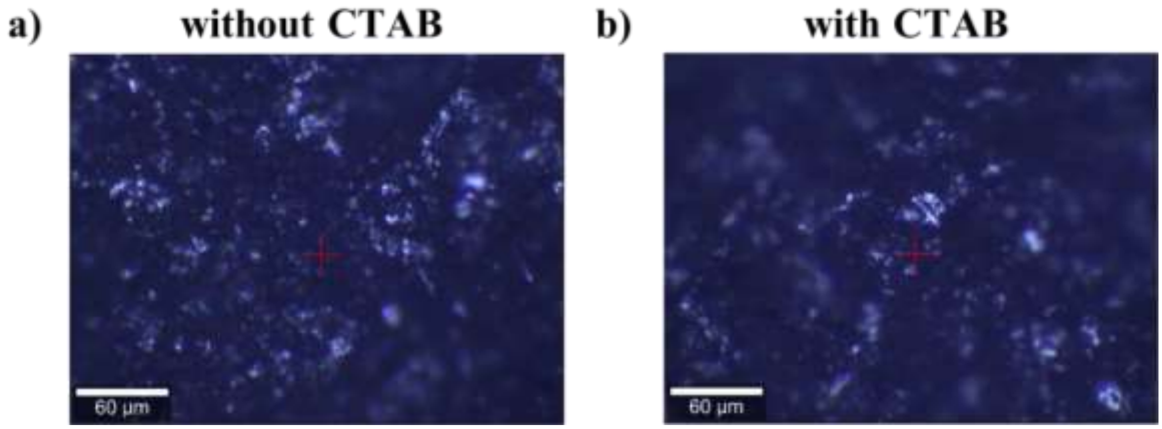

Fig. S1. Optical Raman micrographs of the SPE CB-G surface. **a**) Unmodified electrode (without CTAB); **b**) electrode modified with CTAB. Images were recorded using a WITec alpha300 RA confocal Raman microscope with a 60 µm scale bar. The red cross indicates the region selected for Raman spectral acquisition.

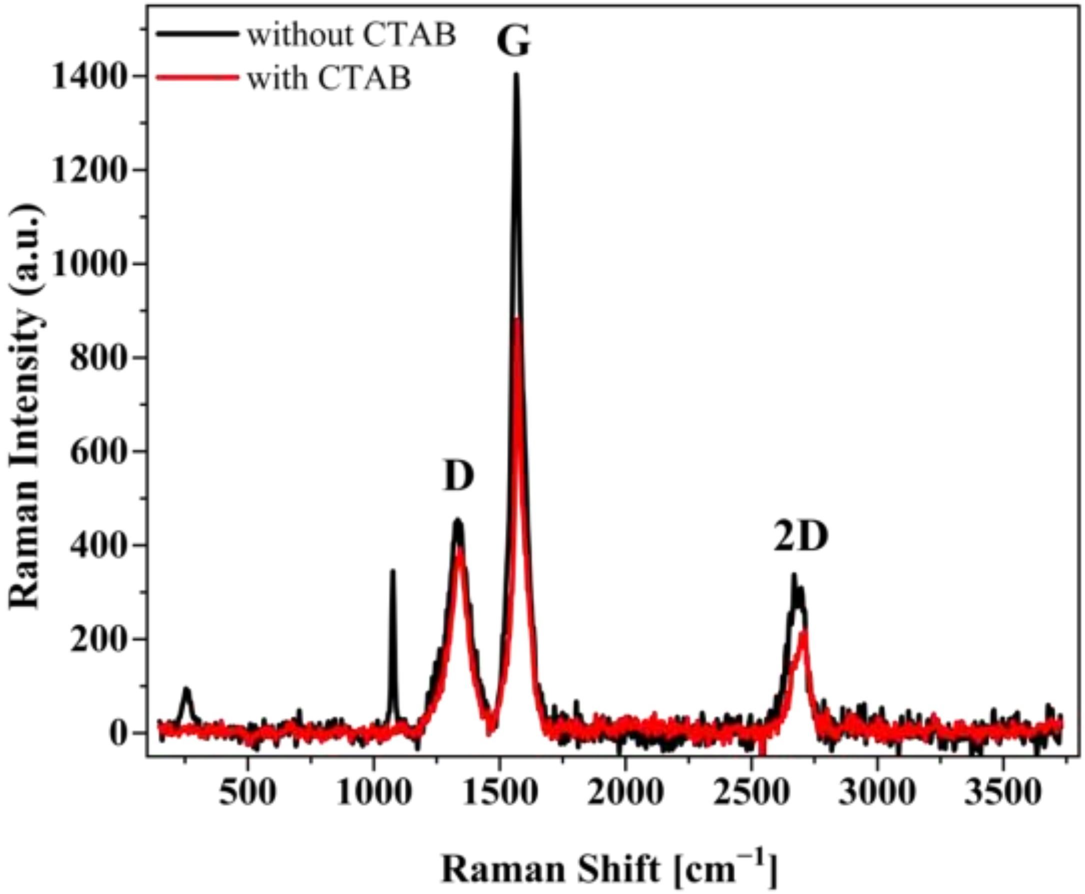

Fig. S2. Raman spectra of the SPE CB-G surface before (black line) and after (red line) *in situ* modification with 80 µmol L^{-1} CTAB.

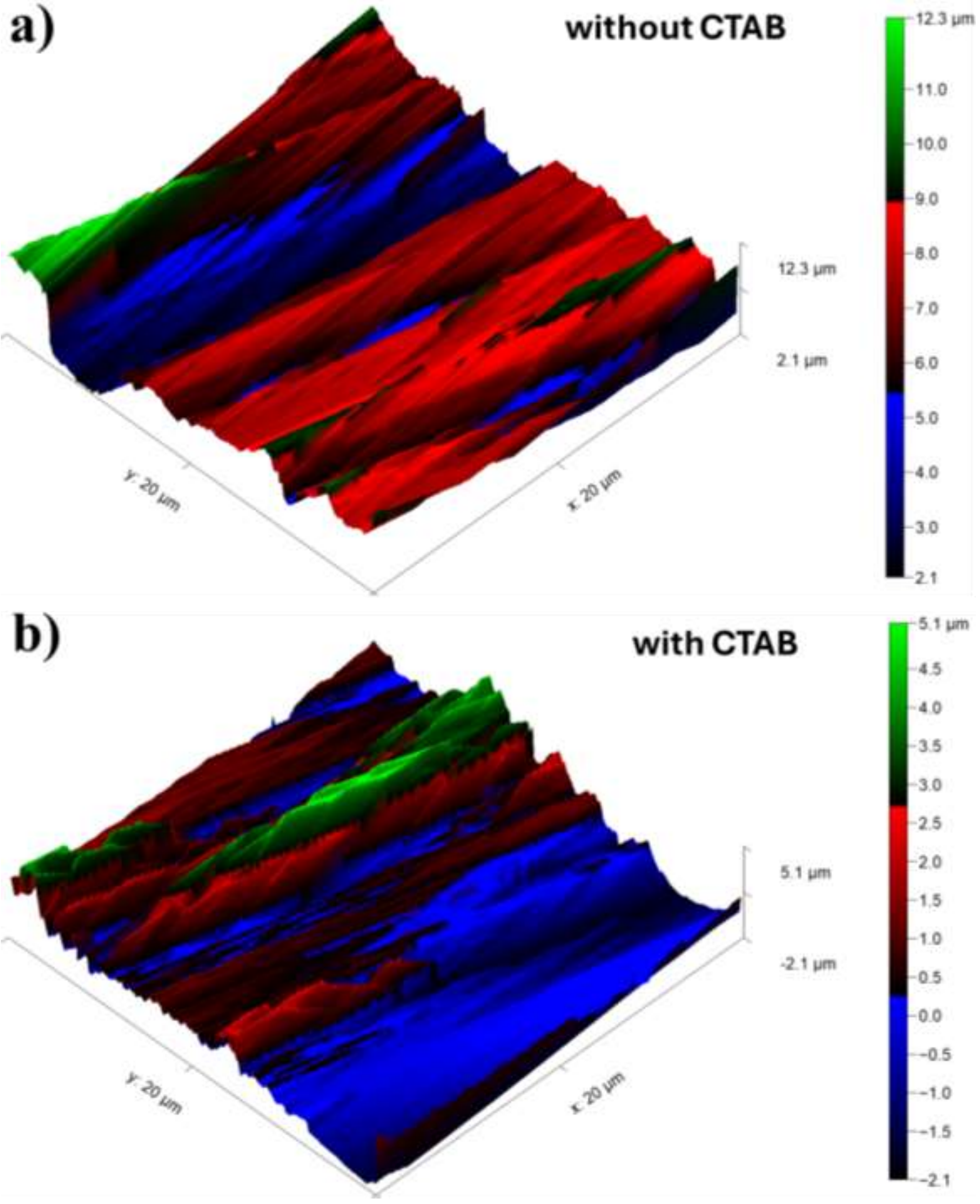

Fig. S3. Representative 3D AFM topographical images of the SPE CB-G surface. **a**) Unmodified electrode (without CTAB); **b**) electrode modified with CTAB. Images were acquired over a 20.0 µm × 20.0 µm area using topography mode. A noticeable reduction in surface roughness is observed after CTAB adsorption, suggesting a more homogeneous and compact film formation.

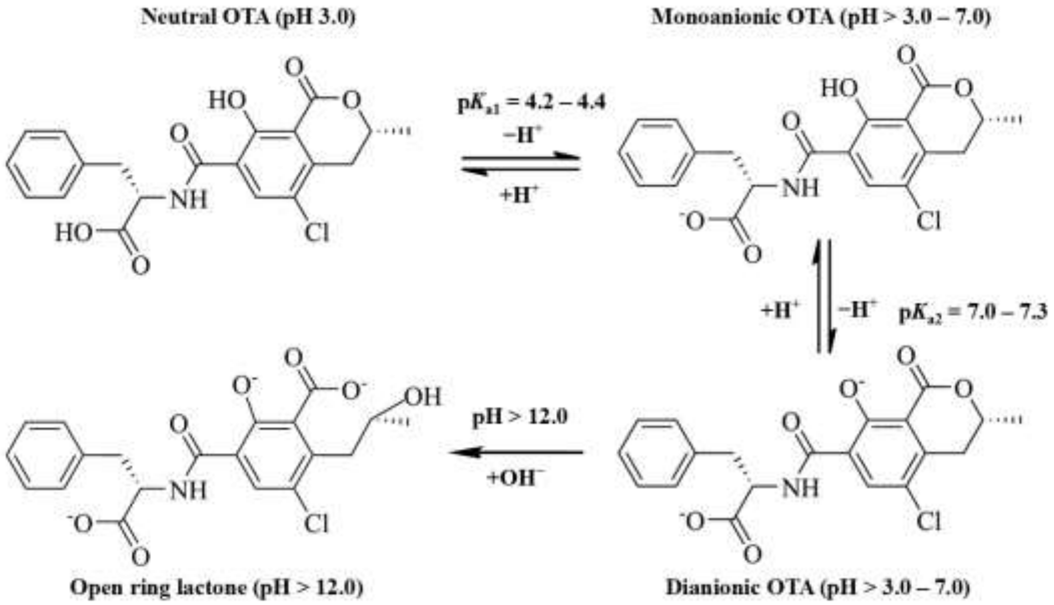

Fig. S4. Scheme of OTA structures in aqueous solutions under acidic to alkaline conditions. Designed and adapted by the authors from references [1,2].

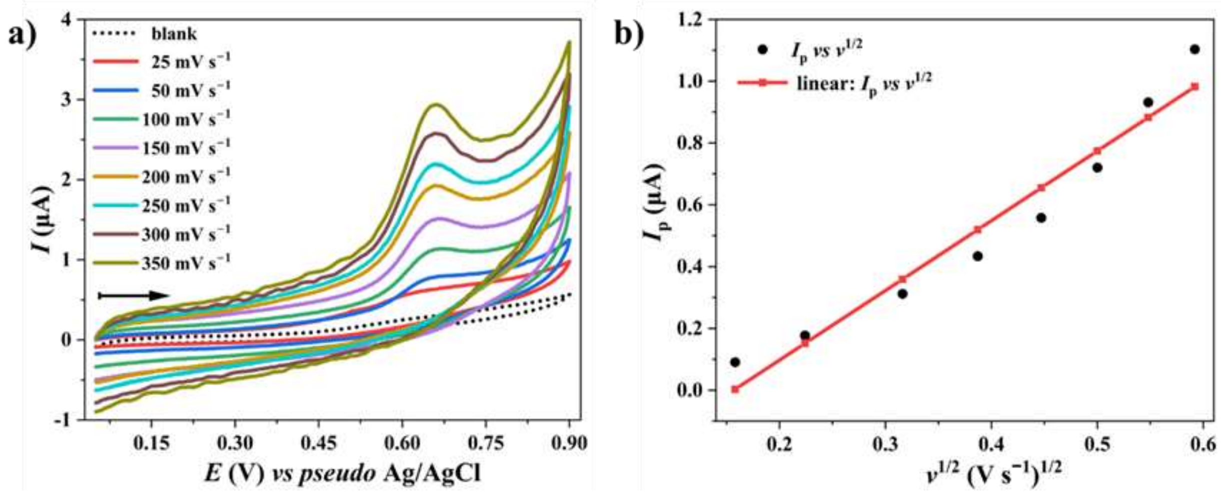

Fig. S5. a) Cyclic voltammograms of OTA (10 µmol L^{-1}) in 0.2 mol L^{-1} PB buffer (pH 7.0) using SPE at different scan rates (25–350 mV s^{-1}). The arrow indicates the direction of the potential scan. **b**) Linear relationship between anodic I_p and the square root of the scan rate ($v^{1/2}$), following the equation I_p (A) = 2.256 × 10^{-6} ($v^{1/2}$) − 3.537 × 10^{-7} (R^2 = 0.947).

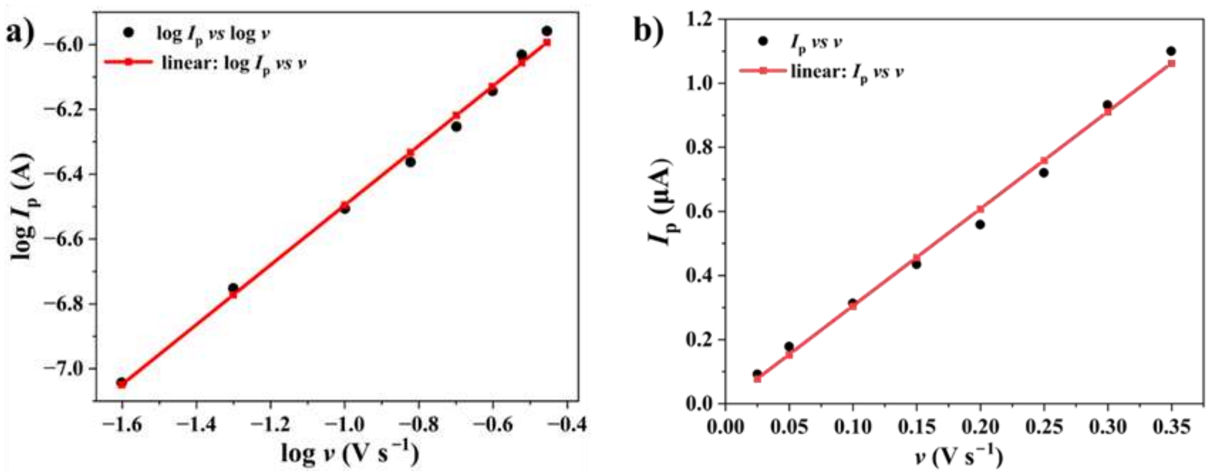

Fig. S6 a) Linear relationship between anodic log I_p and log v follows the equation log I_p (A) = 0.921 (log v) − 5.575 (R^2 = 0.995). **b**) Linear relationship between anodic I_p and v, following the equation I_p (A) = 3.032 × 10^{-6} (v) + 4.421 × 10^{-10} (R^2 = 0.992).

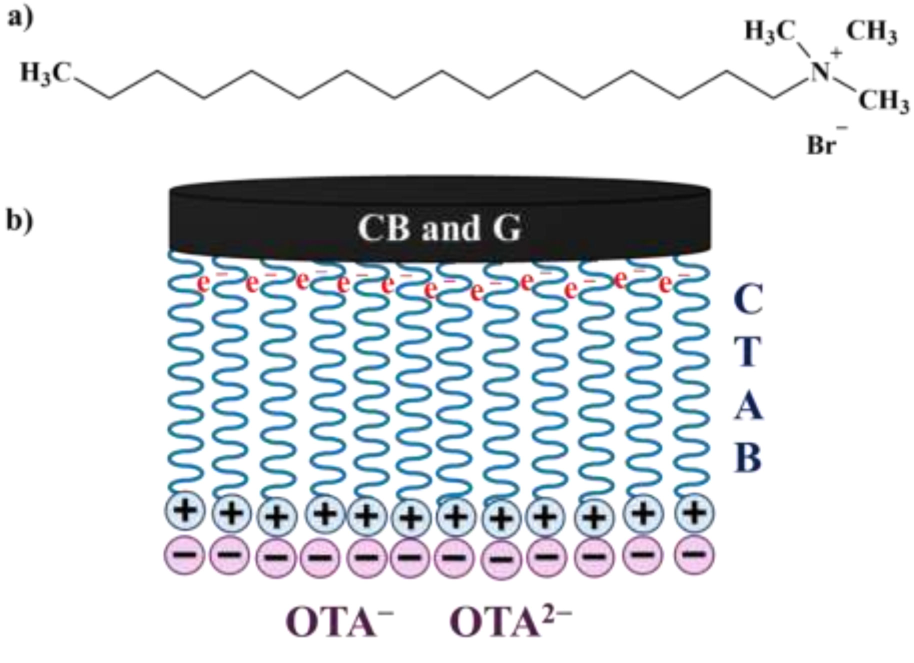

Fig. S7. a) Molecular structure of CTAB, a cationic surfactant with a hydrophobic tail and positively charged polar head. **b**) CTAB interacts with the electrode material (CB – carbon black and G – graphite) and its arrangement on the electrode surface, forming a monolayer with the cationic head oriented towards the solution. The diagram also illustrates the interaction between CTAB and OTA in its monoanionic (OTA^{-}) and dianionic (OTA^{2-}) forms in a 0.2 mol L^{-1} PB buffer (pH 7.0). Designed by the authors from references [3,4].

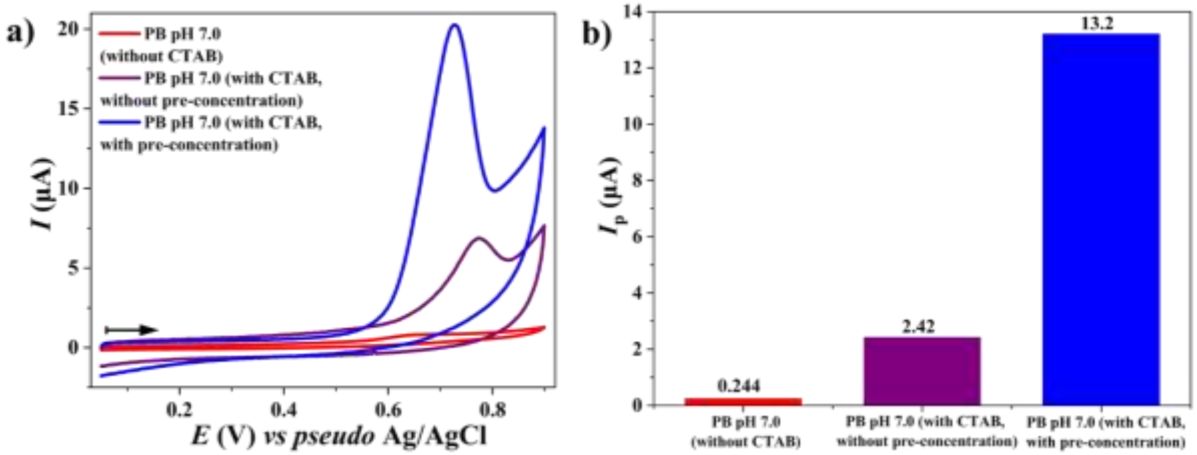

Fig. S8. a) Cyclic voltammograms of OTA oxidation in 0.2 mol L^{-1} PB buffer (pH 7.0) under three conditions: without CTAB (red), with CTAB without pre-concentration (purple), and with CTAB with pre-concentration (blue). The concentrations of OTA and CTAB were 10 and 80 µmol L^{-1}, respectively. The arrow indicates the scan direction. Scan rate: 50 mV s^{-1}; step potential: 2 mV. With CTAB, the deposition parameters were E_{dep} = +0.55 V, t_{dep} =205 s, and the stirring rate = 500 rpm. **b**) Bar graph comparing the anodic peak current (I_p) for OTA oxidation under the same conditions as those in **Fig. a**, highlighting the highest I_p for the system with CTAB and pre-concentration.

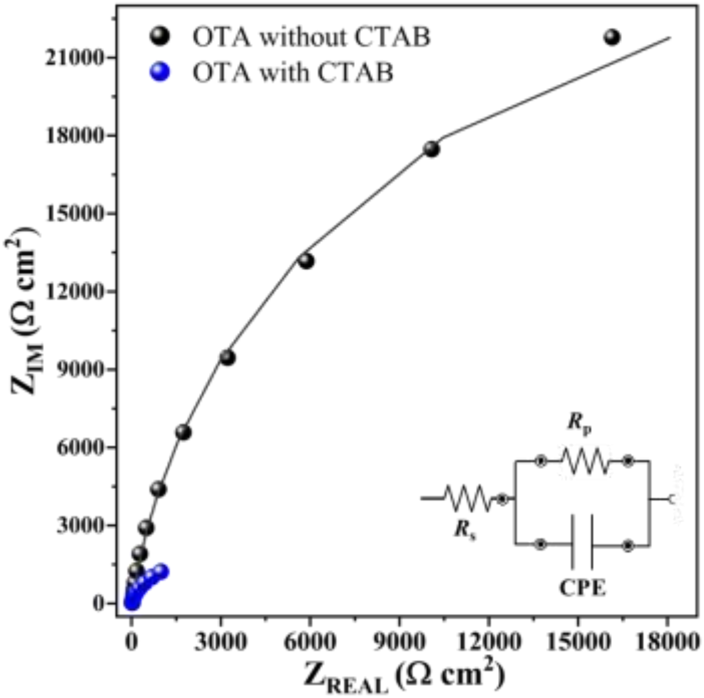

Fig. S9. Impedance diagrams for the SPE CB-G in 0.2 mol L^{-1} PB buffer (pH 7.0) containing 25 µmol L^{-1} OTA. The black circles represent measurements in the absence of CTAB, and blue circles correspond to measurements in the presence of 80 µmol L^{-1} CTAB. The solid lines represent fits based on the Randles equivalent circuit in the insert.

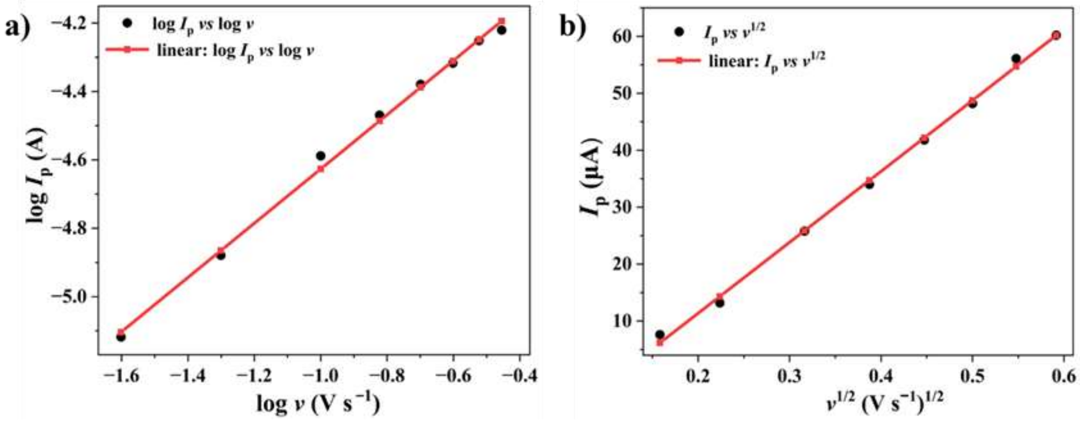

Fig. S10. a) The linear relationship between anodic log I_p and log v follows the equation log I_p (A) = 0.793 (log v) – 3.833 (R^2 = 0.996). **b)** Linear relationship between anodic I_p and $v^{1/2}$, following the equation I_p (A) = 1.246 × 10^{-4} ($v^{1/2}$) − 1.353 × 10^{-5} (R^2 = 0.997).

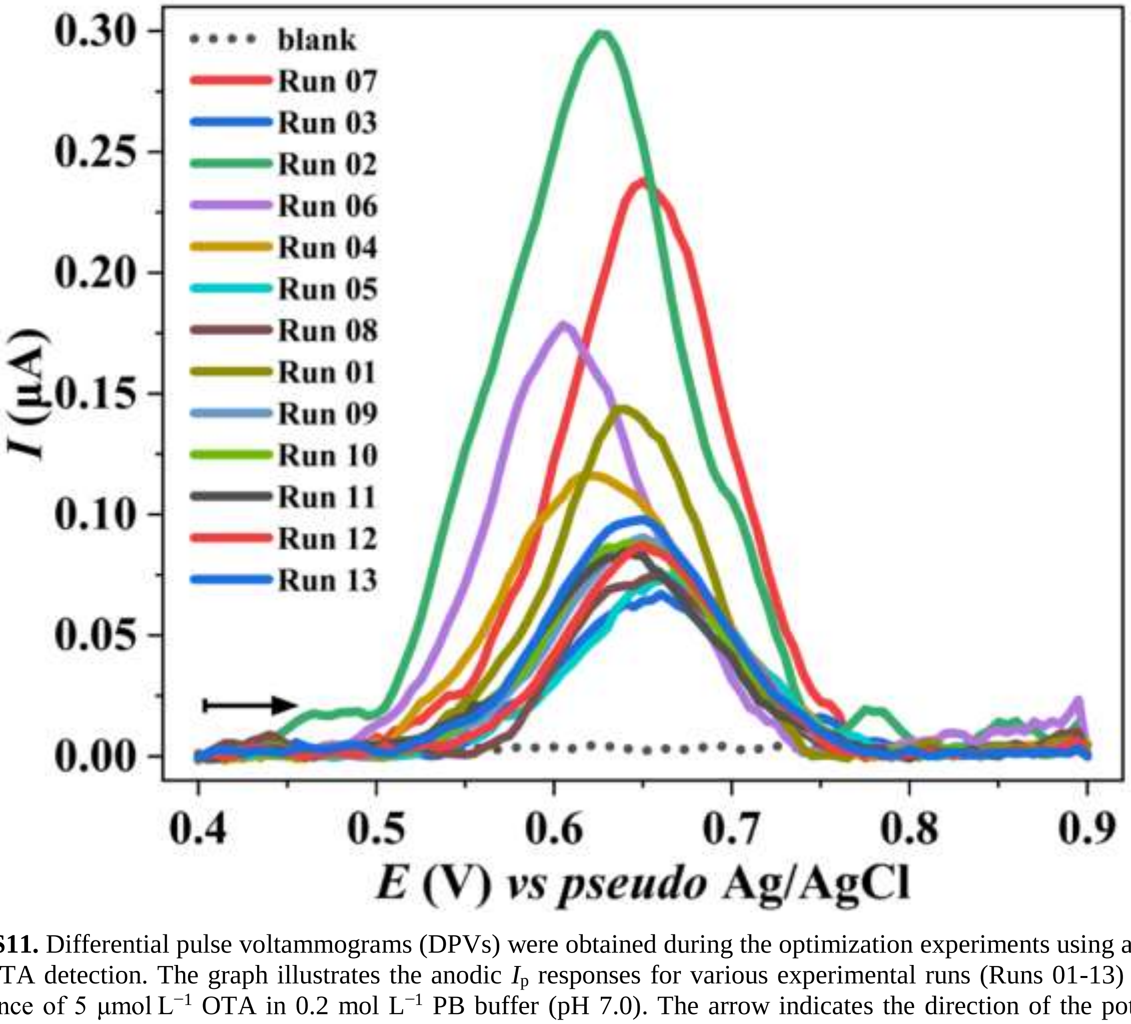

Fig. S11. Differential pulse voltammograms (DPVs) were obtained during the optimization experiments using a CCD for OTA detection. The graph illustrates the anodic I_p responses for various experimental runs (Runs 01-13) in the presence of 5 μmol L^{-1} OTA in 0.2 mol L^{-1} PB buffer (pH 7.0). The arrow indicates the direction of the potential scanning. The experimental conditions were a scan rate of 10 mV s^{-1} and a step potential of 5 mV. The parameters investigated included the pulse potential (E_{pulse}, mV) and pulse time (t_{pulse}, ms), highlighting their impact on I_p.

Table S4. Results of the CCD were conducted to optimize the DPV parameters (with the center point highlighted in bold) using SPE in 0.2 mol L^{-1} PB buffer (pH 7.0).

Run#	Pulse potential E_{pulse}, mV (X_1)	Pulse time t_{pulse}, ms (X_2)	X_1	X_2	I_p (μA)	E_p (V)
01	50	25	−1	−1	0.142	0.645
02	100	25	1	−1	0.286	0.630
03	50	75	−1	1	0.067	0.660
04	100	75	1	1	0.115	0.625
05	(39.65) 40	50	−1.41	0	0.073	0.670
06	(110.35) 110	50	1.41	0	0.175	0.610
07	75	(14.65) 15	0	−1.41	0.235	0.655
08	75	(85.35) 85	0	1.41	0.073	0.660
**09**	**75**	**50**	**0**	**0**	**0.090**	**0.650**
**10**	**75**	**50**	**0**	**0**	**0.085**	**0.655**
**11**	**75**	**50**	**0**	**0**	**0.084**	**0.645**
**12**	**75**	**50**	**0**	**0**	**0.088**	**0.650**
**13**	**75**	**50**	**0**	**0**	**0.097**	**0.650**

Table S5. The coded coefficients were derived from the quadratic model obtained through 2^2 CCD for the optimization of the DPV parameters.

Parameter	Value	Standard Error	95% LCL	95% UCL	t-Value	Prob > |t|
(Intercept)	0.089	0.005	0.076	0.100	18.667	3.142E−7
E_{pulse}	0.042	0.004	0.033	0.051	11.176	1.023E−5
t_{pulse}	−0.059	0.004	−0.068	−0.050	−15.792	9.894E−7
$E_{pulse}\times E_{pulse}$	0.021	0.004	0.011	0.030	5.201	0.001
$t_{pulse}\times t_{pulse}$	0.036	0.004	0.026	0.045	8.920	4.519E−5
$E_{pulse}\times t_{pulse}$	−0.024	0.005	−0.036	−0.011	−4.513	0.003

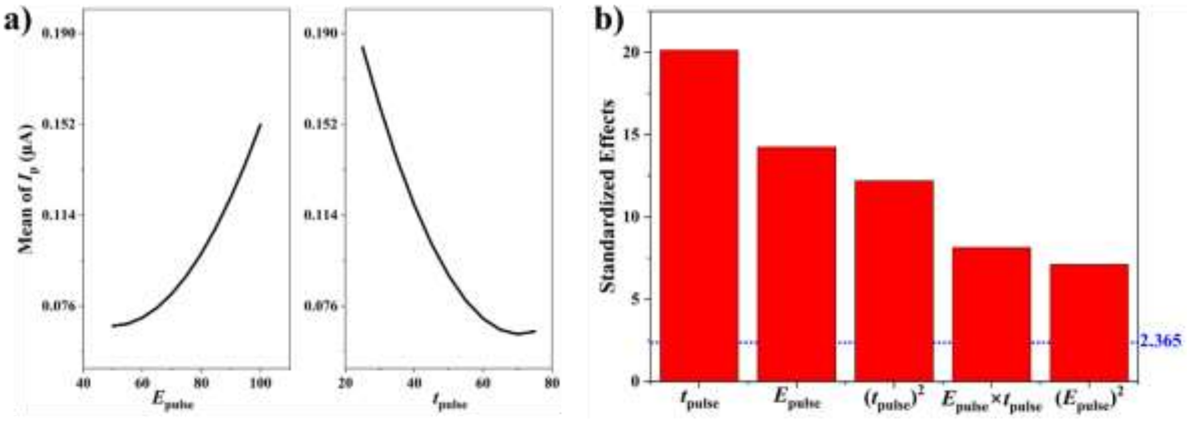

Fig. S12. **a**) Main effects of E_{pulse} and t_{pulse} on the peak current (I_p) in DPV analysis. The positive effect of E_{pulse} and the negative effect of t_{pulse} are depicted, showing that increasing E_{pulse} enhances I_p, whereas increasing t_{pulse} reduces it. **b**) Pareto chart of standardized effects for I_p, highlighting the statistical significance of t_{pulse}, E_{pulse}, and their quadratic and interaction terms. The critical value (2.365) at the 95% confidence level is marked by the dashed blue line, emphasizing the dominant effect of t_{pulse}.

Table S6. ANOVA results for the quadratic model obtained from a 2^2 CCD for the optimization of DPV parameters in the determination of OTA using SPE CB-G.

Source	DF	Sum of Squares	Mean Square	F Value	Prob > F
E_{pulse}	1	0.014	0.014	124.91	1.023E−5
t_{pulse}	1	0.028	0.028	249.37	9.894E−7
$E_{pulse} \times E_{pulse}$	1	0.002	0.002	16.58	0.005
$t_{pulse} \times t_{pulse}$	1	0.009	0.009	79.57	4.519E−5
$E_{pulse} \times t_{pulse}$	1	0.002	0.002	20.36	0.003
Error	7	7.920E−4	1.131E−4	–	–
Lack of fit	3	6.852E−4	2.284E−4	8.55	0.032
Pure Error	4	1.068E−4	2.67E−5	–	–
Total	12	0.056	–	–	–

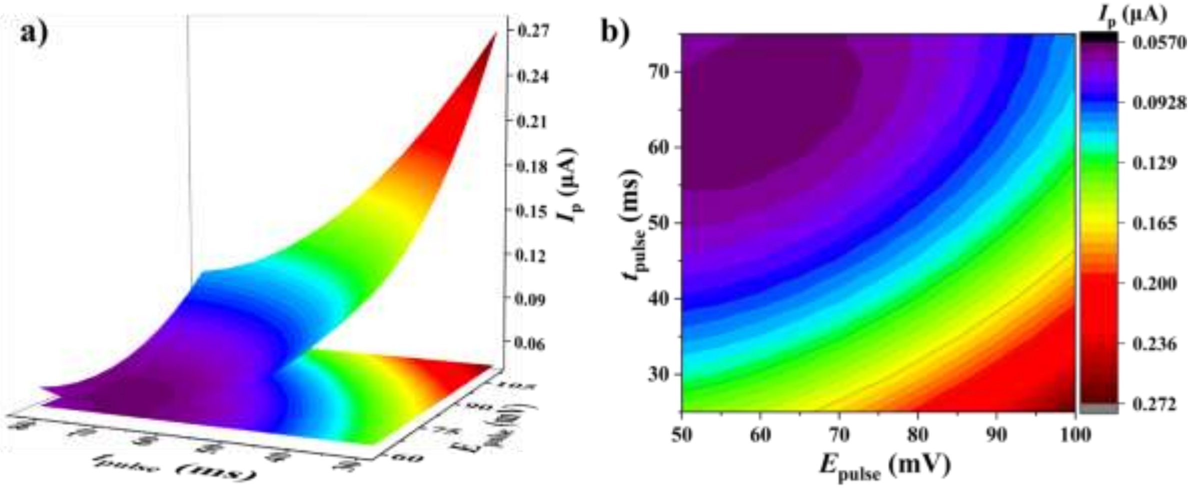

Fig. S13. **a**) Response surface plot illustrating the interaction between E_{pulse} and t_{pulse} on I_p in DPV analysis. The plot demonstrates how changes in these parameters influence the sensor response. **b**) Contour plot showing the relationship between E_{pulse}, t_{pulse}, and I_p, providing a detailed visualization of the response surface. The gradient highlights regions of optimized sensor performance, with higher I_p values represented by warmer colors.

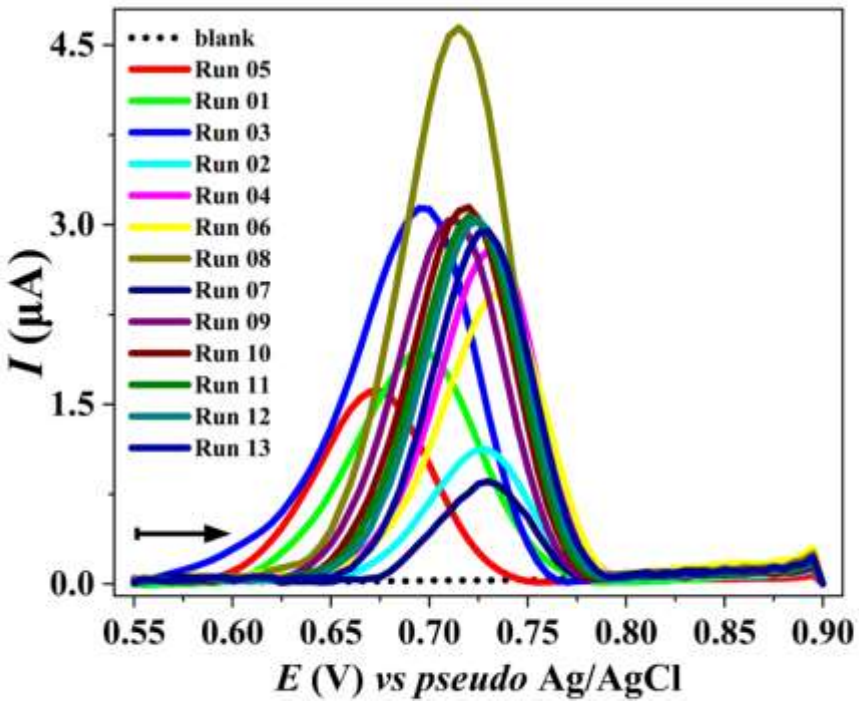

Fig. S14. Adsorptive stripping differential pulse voltammograms (AdSDPVs) obtained during the optimization experiments using a CCD to evaluate the effects of deposition time (t_{dep}, s) and CTAB concentration (µmol L^{-1}) on the anodic I_p in the presence of 201.91 ng mL^{-1} (0.5 µmol L^{-1}) OTA in 0.2 mol L^{-1} PB buffer (pH 7.0). The AdSDPV method involves applying a fixed deposition potential (E_{dep} = +0.55 V) to accumulate the analyte on the working electrode before performing the DPV scan. The arrow indicates the direction of the potential scanning. Experimental conditions: scan rate of 10 mV s^{-1}, step potential of 5 mV.

Table S7. The results of the CCD were conducted to optimize the AdSDPV parameters (with the center point highlighted in bold) using SPE in 0.2 mol L^{-1} PB buffer (pH 7.0) containing CTAB.

Run#	CTAB µmol L^{-1} (X_1)	Deposition time t_{dep}, s (X_2)	X_1	X_2	I_p (µA)	E_p (V)
01	40	60	−1	−1	1.911	0.700
02	120	60	1	−1	1.087	0.730
03	40	180	−1	1	3.110	0.700
04	120	180	1	1	2.687	0.735
05	(23.43) 24	120	−1.41	0	1.596	0.675
06	(136.57) 137	120	1.41	0	2.378	0.735
07	80	(35.14) 35	0	−1.41	0.824	0.730
08	80	(204.86) 205	0	1.41	4.581	0.715
**09**	**80**	**120**	**0**	**0**	2.987	**0.715**
**10**	**80**	**120**	**0**	**0**	3.099	**0.720**
**11**	**80**	**120**	**0**	**0**	2.994	**0.725**
**12**	**80**	**120**	**0**	**0**	2.994	**0.725**
**13**	**80**	**120**	**0**	**0**	2.884	**0.730**

Table S8. Coded coefficients were derived from the quadratic model obtained using a 2^2 CCD to optimize the AdSDPV parameters in the presence of CTAB.

Parameter	Value	Standard Error	95% LCL	95% UCL	*t*-Value	Prob > |*t*|
(Intercept)	2.992	0.210	2.494	3.489	14.225	2.016E−6
CTAB	−0.018	0.166	−0.412	0.375	−0.106	0.918
t_{dep}	1.014	0.166	0.621	1.407	6.099	4.915E−4
CTAB×CTAB	−0.539	0.178	−0.960	−0.117	−3.022	0.019
$t_{dep}\times t_{dep}$	−0.181	0.178	−0.603	0.241	−1.015	0.344
CTAB×t_{dep}	0.100	0.235	−0.456	0.656	0.426	0.683

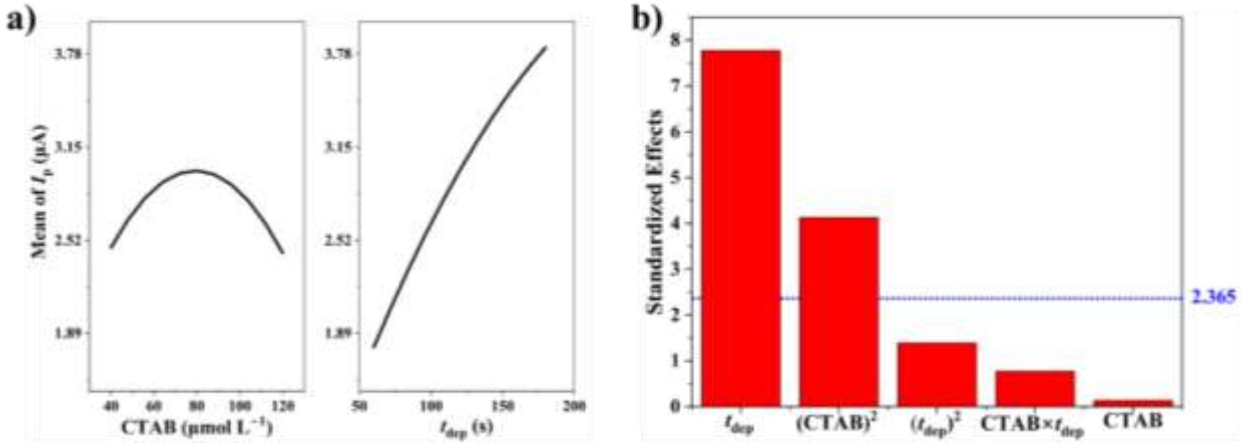

Fig. S15. a) Main effects plot illustrating the influence of CTAB concentration (μmol L^{-1}) and deposition time (t_{dep}) on anodic I_p in AdSDPV analysis. The plot shows that, while t_{dep} positively affects I_p, the effect of CTAB is not statistically significant. **b**) Pareto chart of standardized effects for I_p, demonstrating the lack of statistical significance of CTAB, with its bar falling below the critical t-value threshold (2.365), whereas t_{dep} exhibited a strong and statistically significant effect.

Table S9. ANOVA results for the quadratic model obtained through a 2^2 CCD to optimize the AdSDPV parameters for the determination of OTA using SPE CB-G in the presence of CTAB.

Source	DF	Sum of Squares	Mean Square	F Value	Prob > F
CTAB	1	0.002	0.002	0.011	0.918
t_{dep}	1	8.226	8.226	37.20	4.915E−4
CTAB×CTAB	1	1.878	1.878	8.49	0.022
t_{dep}×t_{dep}	1	0.228	0.228	1.03	0.344
CTAB×t_{dep}	1	0.040	0.040	0.182	0.683
Error	7	1.548	0.221	–	–
Lack of fit	3	1.525	0.508	87.84	4.171E−4
Pure Error	4	0.023	0.006	–	–
Total	12	11.923	–	–	–

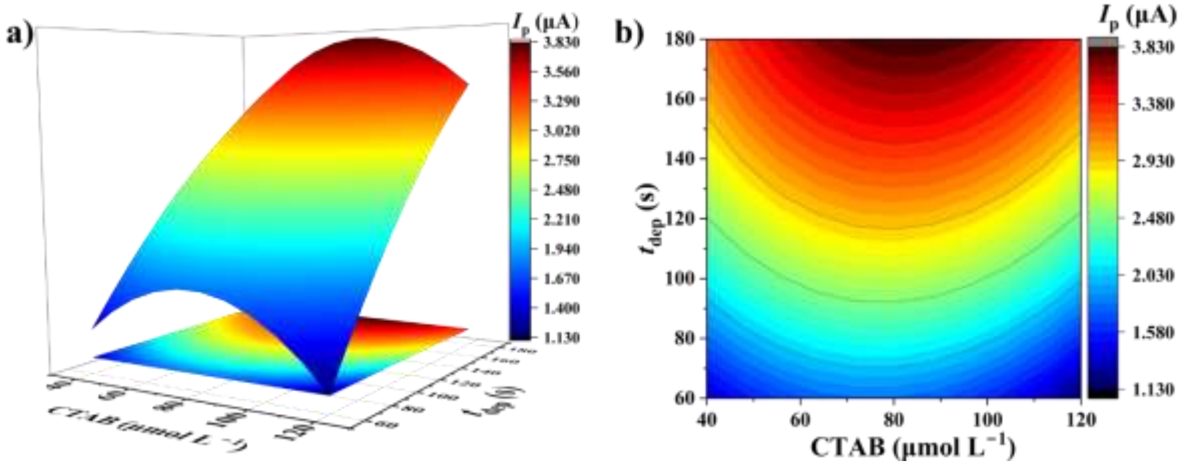

Fig. S16. a) Response surface plot depicting the interaction between CTAB concentration (μmol L^{-1}) and deposition time (t_{dep}) on anodic I_p in AdSDPV analysis. The surface highlights the regions of optimized sensor performance, where I_p increases with specific parameter combinations. **b**) Contour plot providing a two-dimensional representation of the relationship between CTAB concentration and t_{dep}, emphasizing the trends and optimal ranges for maximizing I_p. The warmer colors correspond to higher I_p values.

Table S10. ANOVA results for the regression model describing the calibration curve for OTA determination in 0.2 mol L^{-1} PB buffer (pH 7.0) containing CTAB.

		DF	Sum of Squares	Mean Square	*F* value	Prob > *F*
I_p (μA)	Model	1	29.570	29.570	972.31	< 0.0001
	Error	6	0.183	0.030		
	Total	7	29.752			

Table S11. Intra-electrode stability over consecutive scans and reusability assessment of new batch SPE CB-G (electrode SPE 01 and SPE 02) for OTA detection (201.91 ng mL^{-1}) by AdSDPV.

SPE ID & condition	Scans compared	Initial I_p (µA)	Later I_p (µA)	% Variation in I_p	Notes
SPE 01 (08-May- new)	1st *vs* 5th	4.721	4.301	−8.89	
	1st *vs* 10th	4.721	3.749	−20.59	
	1st *vs* 15th	4.721	3.298	−30.14	
	1st *vs* 20th	4.721	3.843	−18.60	
	1st *vs* 25th	4.721	2.354	−50.14	
	1st *vs* 30th	4.721	1.985	−57.95	End of initial use
SPE 01 (09-May - Reused)	Initial 1st *vs* Reuse 1st	4.721	3.394	−28.11	Inter-day reuse
	Reuse 1st *vs* Reuse 5th	3.394	2.968	−12.55	Stability during reuse
SPE 02 (09-May - new)	1st *vs* 5th	5.108	5.350	+4.74	
	1st *vs* 10th	5.108	5.340	+4.54	
	1st *vs* 15th	5.108	5.079	−0.57	
	1st *vs* 20th	5.108	4.464	−12.61	
	1st *vs* 25th	5.108	4.383	−14.19	
	1st *vs* 30th	5.108	3.571	−30.09	End of initial use
SPE 02 (09-May - Reused)	Initial 1st *vs* Reuse 1st	5.108	4.017	−21.36	Intra-day reuse
	Reuse 1st *vs* Reuse 5th	4.017	3.799	−5.43	Stability during reuse

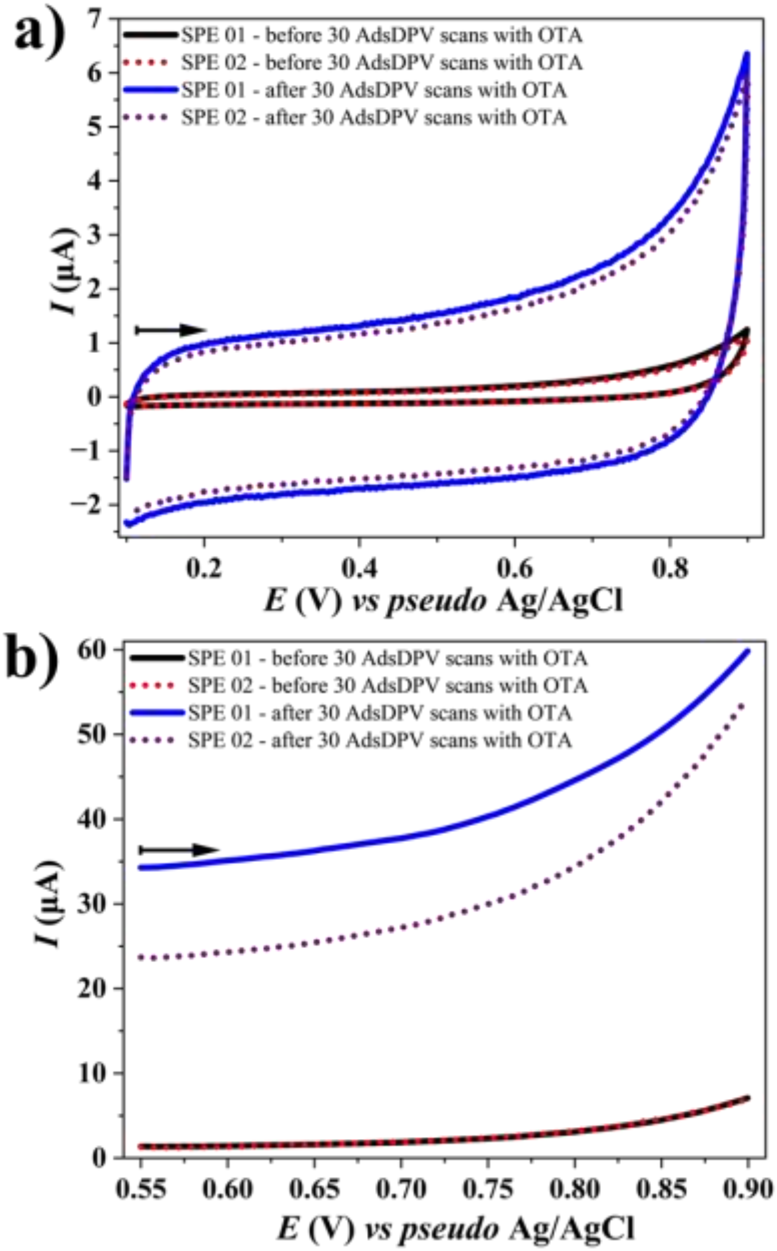

Fig. S17. a) Cyclic voltammograms of SPE 01 and SPE 02 before and after 30 consecutive AdSDPV runs for OTA detection, followed by washing. Conditions: 0.2 mol L^{-1} PB buffer (pH 7.0) containing 80 µmol L^{-1} CTAB; scan rate: 50 mV s^{-1}; step potential: 2 mV. Small scan window (black line and red points): voltammograms recorded before AdSDPV application. Large scan window (blue line and purple points): voltammograms recorded after 30 AdSDPV cycles. A marked increase in background capacitive current is observed upon electrode reuse. **b**) Blank AdSDPV responses of the same electrodes under the same conditions (E_{pulse} = 100 mV, t_{pulse} = 25 ms, E_{dep} = +0.55 V, t_{dep} = 205 s, and stirring rate = 500 rpm. Scan rate: 10 mV s^{-1}; step potential: 5 mV), showing current baselines before (black line and red points: lower background) and after (blue line and purple points: elevated background) 30 AdSDPV runs. The results highlight a substantial increase and fluctuation in background current after repeated use, indicating reduced signal stability and limited electrode reusability.

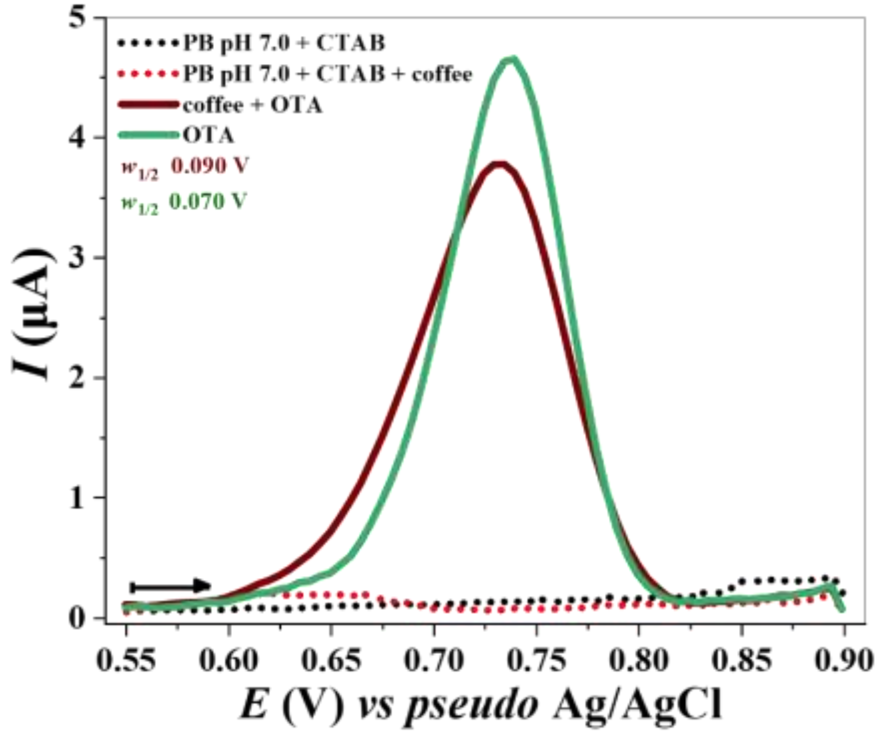

Fig. S18. Adsorptive stripping differential pulse voltammograms (AdSDPV) obtained using SPE CB-G in the presence of OTA (210.0 ng mL^{-1}) in 0.2 mol L^{-1} PB buffer (pH 7.0) modified with CTAB (80 µmol L^{-1}). The voltammograms compare blank 0.2 mol L^{-1} PB buffer (pH 7.0) with CTAB (dotted black line), blank coffee in 0.2 mol L^{-1} PB buffer (pH 7.0) with CTAB (dotted red line), OTA standard (solid green line) in 0.2 mol L^{-1} PB buffer (pH 7.0) with CTAB, and OTA in the coffee matrix (solid brown line) in 0.2 mol L^{-1} PB buffer (pH 7.0) with CTAB. The oxidation peak of OTA in the coffee matrix had a width at half height ($W_{1/2}$) of 0.090 V, which was larger than the standard OTA $W_{1/2}$ of 0.070 V, suggesting the influence of the matrix on the electron transfer process. Experimental conditions: deposition potential (E_{dep}) = +0.55 V; deposition time (t_{dep}) = 205 s; stirring rate = 500 rpm; pulse amplitude (E_{pulse}) = 100 mV; pulse time (t_{pulse}) = 25 ms; step potential = 5 mV; scan rate = 10 mV s^{-1}. The arrow indicates the scan direction.

Table S12. ANOVA results for the regression model describing the calibration curve for OTA determination in a coffee matrix using 0.2 mol L^{-1} PB buffer (pH 7.0) with CTAB.

		DF	Sum of Squares	Mean Square	*F* value	Prob > *F*
I_p (µA)	Model	1	10.138	10.138	666.44	< 0.0001
	Error	4	0.061	0.015		
	Total	5	10.199			

References

(1) Cagnasso, I.; Tonachini, G.; Berto, S.; Giacomino, A.; Mandrile, L.; Maranzana, A.; Durbiano, F. Comprehensive Study on the Degradation of Ochratoxin A in Water by Spectroscopic Techniques and DFT Calculations. *RSC Adv.* **2019**, *9* (34), 19844–19854. https://doi.org/10.1039/C9RA02086A.

(2) Dohnal, V.; Pavlikova, L.; Kuca, K. The pH and Mobile Phase Composition Effects Ochratoxin A Fluorescence at Liquid Chromatography. *J. Chromatogr. Sci.* **2010**, *48* (9), 766–770. https://doi.org/10.1093/chromsci/48.9.766.
(3) Sultana, A.; Sazawa, K.; Okazaki, T.; Islam, Md. S.; Hata, N.; Sugawara, K.; Kuramitz, H. Adsorptive Voltammetry for the Determination of Ochratoxin A Using Enrichment Effect by Cationic Surfactants. *Electroanalysis* **2018**, *30* (10), 2265–2272. https://doi.org/10.1002/elan.201800226.
(4) Maloku, A.; Berisha, L.; Jashari, G.; Arbneshi, T.; Kalcher, K. Enhancement Effect of Cetyltrimethylammonium Bromide on Electrochemical Determination of Chlorophenols Using a Carbon Paste Electrode. *J. Anal. Chem.* **2020**, *75* (3), 358–365. https://doi.org/10.1134/S1061934820030120.
